# Evolution of a SHOOTMERISTEMLESS transcription factor binding site promotes fruit shape determination

**DOI:** 10.1038/s41477-024-01854-1

**Published:** 2024-12-12

**Authors:** Zhi-Cheng Hu, Mateusz Majda, Hao-Ran Sun, Yao Zhang, Yi-Ning Ding, Quan Yuan, Tong-Bing Su, Tian-Feng Lü, Feng Gao, Gui-Xia Xu, Richard S. Smith, Lars Østergaard, Yang Dong

**Affiliations:** 1 State Key Laboratory of Plant Diversity and Specialty Crops, Institute of Botany, Chinese Academy of Sciences, Beijing, China; 2 University of Chinese Academy of Sciences, Beijing, China; 3 China National Botanical Garden, Beijing, China; 4 Department of Plant Molecular Biology, University of Lausanne, Lausanne, Switzerland; 5 Computational and Systems Biology Department, John Innes Centre, Norwich, UK; 6 Beijing Vegetable Research Center, Beijing Academy of Agriculture and Forestry Sciences, Beijing, China; 7 Crop Genetics Department, John Innes Centre, Norwich, UK; 8 Department of Biology, University of Oxford, Oxford, UK

**Keywords:** Plant development, Plant evolution

## Abstract

In animals and plants, organ shape is primarily determined during primordium development by carefully coordinated growth and cell division^1–3^. Rare examples of post-primordial change in morphology (reshaping) exist that offer tractable systems for the study of mechanisms required for organ shape determination and diversification. One such example is morphogenesis in *Capsella* fruits whose heart-shaped appearance emerges by reshaping of the ovate spheroid gynoecium upon fertilization^4^. Here we use whole-organ live-cell imaging and single-cell RNA sequencing (scRNA-seq) analysis to show that *Capsella* fruit shape determination is based on dynamic changes in cell growth and cell division coupled with local maintenance of meristematic identity. At the molecular level, we reveal an auxin-induced mechanism that is required for morphological alteration and ultimately determined by a single *cis*-regulatory element. This element resides in the promoter of the *Capsella rubella SHOOTMERISTEMLESS*^5^ (*CrSTM*) gene. The CrSTM meristem identity factor positively regulates its own expression through binding to this element, thereby providing a feed-forward loop at the position and time of protrusion emergence to form the heart. Independent evolution of the STM-binding element in *STM* promoters across Brassicaceae species correlates with those undergoing a gynoecium-to-fruit shape change. Accordingly, genetic and phenotypic studies show that the STM-binding element is required to facilitate the shape transition and suggest a conserved molecular mechanism for organ morphogenesis.

## Abstract (web summary)

This study identifies a molecular mechanism promoting fruit shape variation. Local meristem identity is maintained through autoregulatory activation of the *STM* gene to allow post-fertilization changes in fruit morphology.

## Main

Multicellular organisms produce organs with specific functions for resource acquisition and reproduction^6^. Evolution of such specialized functions is intricately linked with organ morphology diversification that is adaptive in distinct ecological niches^7–9^. For example, evolutionary diversification of fruit shape has led to diverse dispersal strategies adapted to optimizing the success of the next generation^4,10,11^. Such phenotypic diversity requires modulation in the coordination of cell division and growth in reproductive structures^1–3^. However, the molecular mechanism underlying this is poorly understood.

**Fig. 1:**
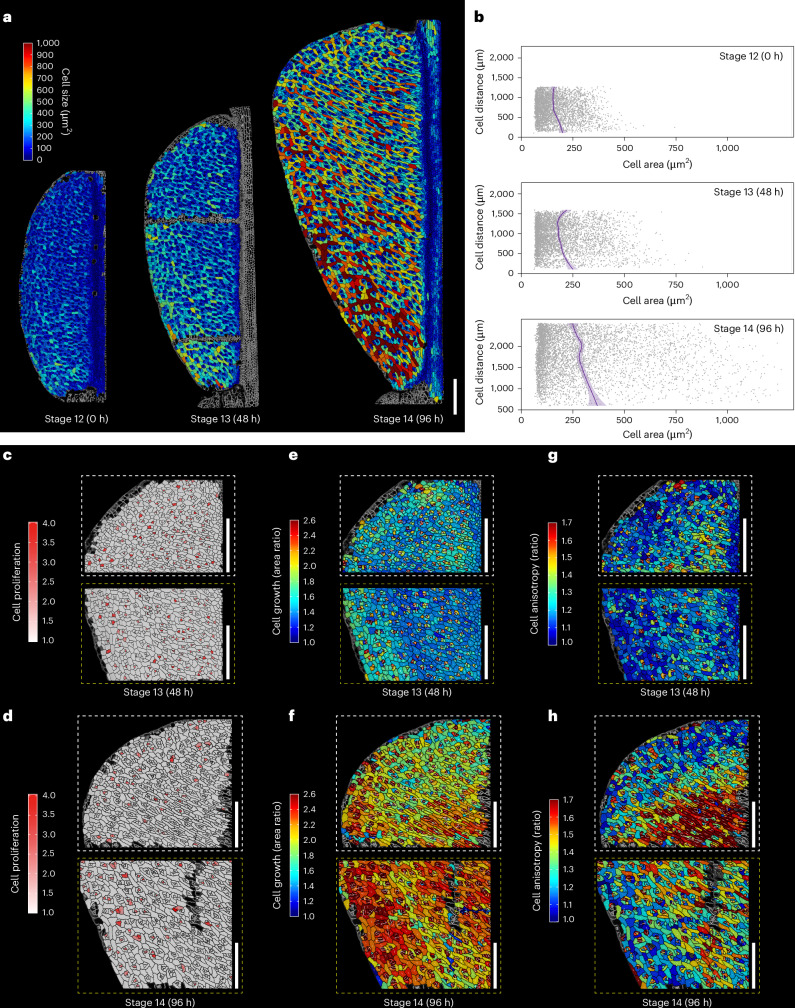
Cellular basis of fruit shape determination in *Capsella.* **a**, Heatmaps depicting cell size in the *Capsella* fruit during 96 h of time-lapse live-cell imaging analysis, corresponding to developmental stages 12, 13 and 14 (0, 48 and 96 h, respectively). **b**, Scatter plots representing cell size distribution along the vertical axes of the fruits at the time points shown in **a**. The *y* axis denotes the inter-cellular distance from the fruit base (0 μm) to the fruit apex. Each dot represents an individual cell. Average cell sizes and confidence intervals are depicted by purple lines and shading, respectively. **c**,**d**, Heatmaps demonstrating cell proliferation of the *Capsella* fruit at the 48 h time point (**c**) and 96 h time point (**d**). **e**,**f**, Heatmaps of cell growth (the rate of cell area increase) of the *Capsella* fruit at the 48 h time point (**e**) and 96 h time point (**f**). **g**,**h**, Heatmaps of cell anisotropy (the ratio of cell expansion in the maximum and minimum principal directions) of the *Capsella* fruit at the 48 h time point (**g**) and 96 h time point (**h**). In **c**–**h**, the white and yellow boxes correspond to the apical regions and basal regions of the valves, respectively, as indicated in the images of the entire valves in Extended Data Fig. 1. Scale bars in **a** and **c**–**h**, 200 μm.

**Extended Data Fig. 1:**
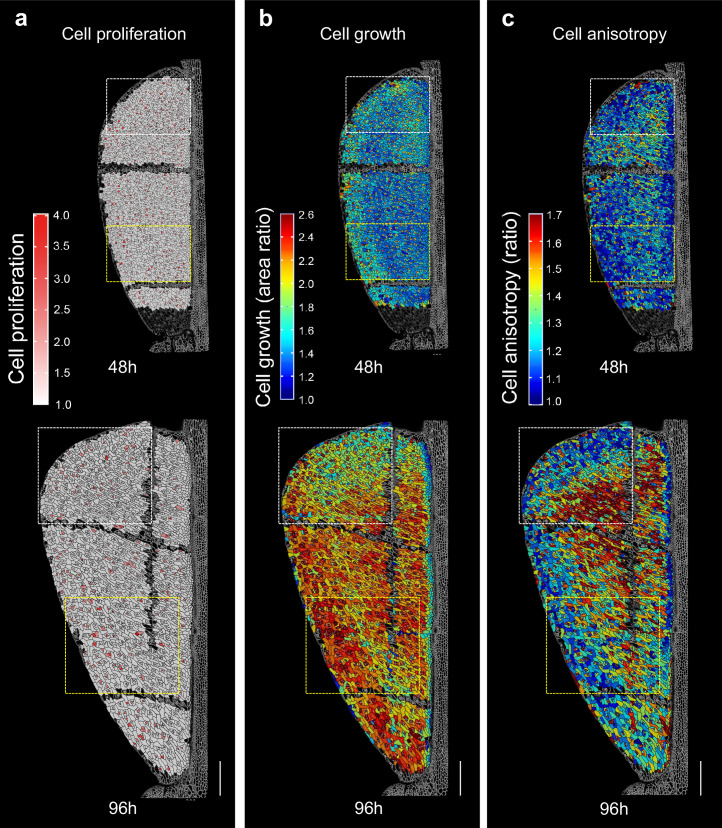
Time-lapse live imaging analysis of *Capsella* fruit development. **a-c**. Heat-maps of valves at 48-hour (48 h) and 96-hour (96 h) time points corresponding to developmental stages 13 and 14, respectively, depicting cell proliferation (**a**), cell growth (**b**) and cell anisotropy (**c**). The white and yellow boxes in a-c indicate the enlarged area shown in Fig. 1 of the main the text. Scale bars in **a** to **c**, 200 μm.

Members of the *Capsella* genus produce fruits that are shaped like a heart^4^. Upon fertilization of the *Capsella* flower, the female reproductive organ undergoes metamorphosis from an oblate spheroid (disc-shaped) gynoecium to a flat, heart-shaped fruit^4,12^. This shape is adopted as the two valves (seed pod walls) each form one half of the heart protruding laterally from the central replum. The *Capsella* fruit therefore provides an excellent system for studying the cellular and molecular bases of organ shape establishment^12^. Using the plasma membrane marker line, *pUBQ10:acyl-YFP*^13^, we performed a large-scale analysis including a total of 18,656 segmented cells of the female reproductive organ from developmental stage 12 (fertilization) to stage 14, during which the heart shape of the fruit gradually emerges^4,12,14^. Using lineage tracking maps, we quantified cell growth parameters that affect form, including growth (the rate of cell area expansion), anisotropy (the direction of cell expansion) and cell proliferation (Fig. 1a and Extended Data Fig. 1a–c). Analysis of cell size revealed a gradient, with larger cells predominantly located at the base of the fruit and progressively smaller cells observed towards the tip (Fig. 1a,b). This suggested a gradient of differentiation from the base to the tip. Such a gradient was not detected in *Arabidopsis* during gynoecium patterning, nor during post-fertilization fruit growth^15,16^, and may reflect the morphological change characterizing *Capsella* fruit morphogenesis. Areal growth rates are initially slightly higher towards the top of the valve near the replum, but increase somewhat at the base between stages 13 and 14 (Fig. 1e,f; shown for the entire valve in Extended Data Fig. 1b). In contrast with post-fertilization growth of the *Arabidopsis* fruit^16^, cells of the *Capsella* fruit continue to divide after fertilization, although at stage 14 cell division is substantially reduced throughout the valve (Fig. 1c,d, Extended Data Fig. 1a and Supplementary Fig. 1). A region with a higher level of anisotropic growth was observed at the apical part of the fruit near the replum at stage 13 growing towards a region of smaller and less anisotropically growing cells at the flank (Fig. 1e,g and Supplementary Fig. 1). This region was greatly expanded at stage 14 (Fig. 1f,h; shown for the entire valve in Extended Data Fig. 1c and Supplementary Fig. 1), suggesting that this area pushes the upper part of the valve outward, thus contributing to creating the heart shape. In summary, organ-wide cellular analysis suggested that coordination of developmentally controlled anisotropic growth and changes in cellular growth and division are required for reshaping of the *Capsella* gynoecium at the onset of fruit development.

**Fig. 2:**
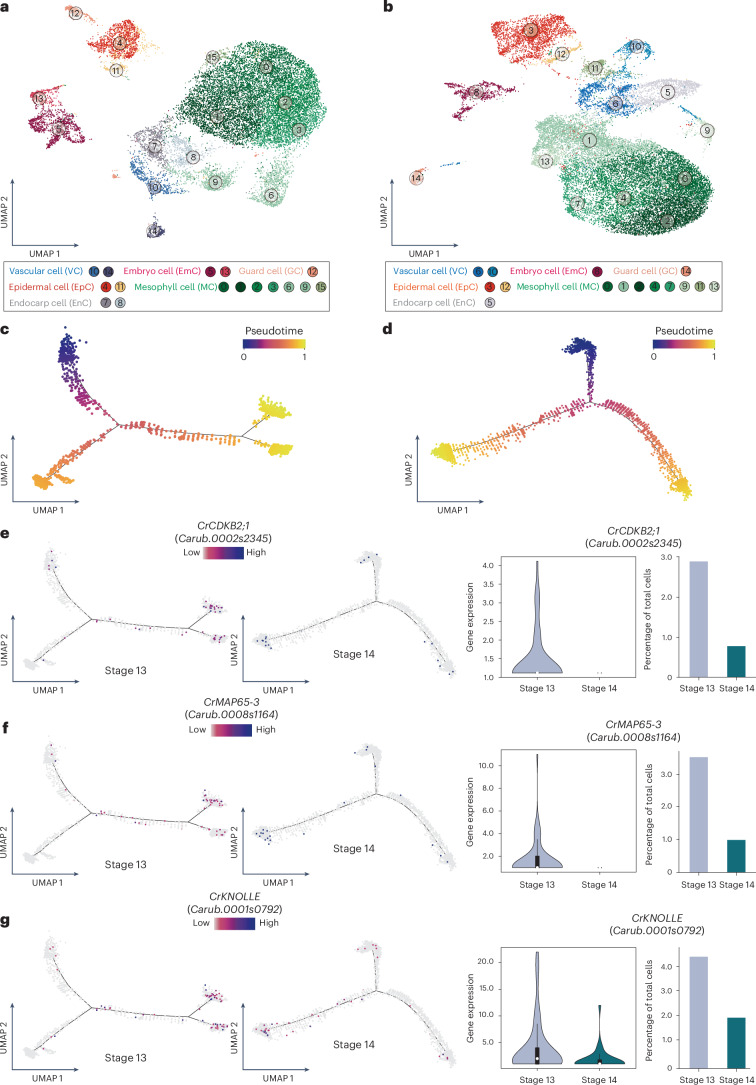
Comparative analysis of cell differentiation trajectories in fruit valve tip epidermal cells. **a**, UMAP visualization of 16 cell clusters in stage 13 fruit valve tips (*n* = 17,453 cells). **b**, UMAP visualization of 15 cell clusters in stage 14 fruit valve tips (*n* = 23,176 cells). In **a** and **b**, each dot denotes a single cell and the colours denote corresponding cell clusters. All of the cell clusters can be grouped into six cell types (see Supplementary Fig. 9) with corresponding colours: vascular cells (clusters 10 and 14 in **a** and 6 and 10 in **b**), embryo cells (clusters 5 and 13 in **a** and 8 in **b**), guard cells (cluster 12 in **a** and 14 in **b**), epidermal cells (clusters 4 and 11 in **a** and 3 and 12 in **b**), mesophyll cells (clusters 0, 1, 2, 3, 6, 9 and 15 in **a** and 0, 1, 2, 4, 7, 9, 11 and 13 in **b**) and endocarp cells (clusters 7 and 8 in **a** and 5 in **b**). **c**,**d**, Monocle 2 analysis showing differentiation trajectories coloured by the pseudotime of the valve tip epidermal cells from stage 13 fruit (**c**; *n* = 1,775 cells; see Methods) and stage 14 fruit (**d**; *n* = 3,000 cells; see Methods). **e**–**g**, Comparisons between stage 13 and stage 14 fruits of the expression patterns (left) and expression levels (middle) of genes involved in the cell cycle and cytokinesis (that is, *CrCDKB2;1* (**e**), *CrMAP65-3* (**f**) and *CrKNOLLE* (**g**)), along with the percentages of cells expressing these genes in valve tip epidermal cells (right). Gene expression levels were calculated using the ggboxplot function (in the ggpubr package in R). Each dot in the differentiation trajectories indicates a single cell. The combines violin and box plots in **e**–**g** (middle) show the data distribution and its probability density. In these box plots, the white circles indicate median values, the edges of the boxes define interquartile ranges, the whiskers represent 95% confidence intervals and the end points indicate minimum and maximum values. The widths of the density plots outside the boxes represent the data frequency.

To characterize the developmental trajectories of the valve tips at the single-cell level, we conducted a comparative scRNA-seq analysis of the valve tips of stage 13 fruits (when shape change initiates) and stage 14 fruits (when shape change is achieved). A total of 32,126 cells from stage 13 valve tips (~260 fruits) and 38,349 cells from stage 14 valves tips (~200 fruits) were isolated and cell walls were removed (Supplementary Fig. 2). These cells were then subjected to scRNA-seq using droplet-based technology on the commercial 10x chromium platform (10x Genomics) (Supplementary Fig. 3). After aligning the data to the *Capsella* genome^17^, removing the low-quality cells and filtering out genes induced during protoplast isolation (see Methods), we obtained gene expression matrices containing 17,979 genes across 17,453 cells for stage 13 samples and 18,449 genes across 23,176 cells for stage 14 samples (see Methods, Supplementary Figs. 4 and 5 and Supplementary Data 1). Data analyses found consistent cell cluster overlap between the two replicates after integration, indicating a high level of experimental reproducibility (Supplementary Fig. 6). We subsequently chose the replicate with more cells captured and used principal component analysis on a gene expression matrix across 2,000 highly variable genes (HVGs). Then, 50 statistically significant principal components (*P* < 0.05) were chosen for in-depth data analysis. These principal components were processed using Seurat, resolving 16 and 15 distinct clusters for stages 13 and 14, respectively (Fig. 2a,b and Supplementary Fig. 7), revealing heterogeneous cell populations. The stage 13 clusters contained 158–3,136 cells and the stage 14 clusters contained 301–4,287 cells (Supplementary Fig. 4). These clusters exhibited a high correlation across the samples and bulk RNA-seq (*ρ* = 0.9273 for stage 13 and *ρ* = 0.9206 for stage 14), demonstrating the reproducibility of the sample (Supplementary Fig. 8).

**Extended Data Fig. 2:**
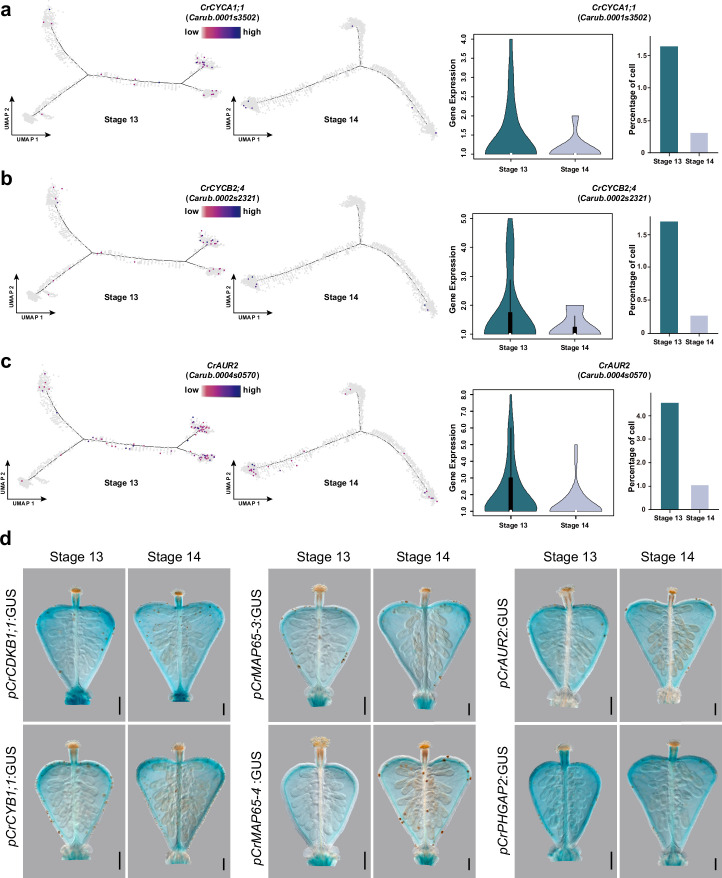
Expression profiles of cell cycle genes in the pseudotime differentiation trajectories and reporter lines. **a**-**c**, Comparisons of the expression patterns, expression levels and percentages of cells expressing the genes involved in cell cycle and cytokinesis (**a**, *CrCYCA1;1*; **b**, *CrCYCB2;4*; **c**, *CrAUR2*) in the valve tip epidermal cells between stage 13 and stage 14 fruits. The gene expression level is calculated by the ggboxplot function (R ggpub r package). Values of gene expression and percentage of cells originate from one of the two single cell sequencing experiments. **d**, Expression patterns of *CrCDKB1;1*, *CrCYCB1;1*, *CrMAP65-3*, *CrMAP65-4*, *CrAUR2* and *CrPHGAP2* by GUS staining with respective reporter lines at developmental stages 13 and 14, showing an even distribution of expression of these genes in the valve cells and a gradual decrease in the expression in the valve tips. In the middle plots of **a**–**c**, the violin plots incorporate box plots to show the data distribution and its probability density. In these box plots, the white circle indicates the median, the edges of the box define the interquartile range, the whiskers represent the 95% confidence interval and the end points indicate the minima and maxima values. The width of the density plot outside the box represents the data frequency. Each dot in the differentiation trajectory of **a** to **c** indicates a single cell. Scale bars in **d**, 100 μm.

The clusters were annotated using orthologues of *Arabidopsis* genes with known biological functions and expression patterns in addition to known markers identified in other scRNA-seq datasets^18^ (Supplementary Data 1 and 2 and Supplementary Fig. 9). Based on this analysis, we grouped the clusters into six distinct cell types at both developmental stages (Fig. 2a,b), consistent with the anatomical structures of the fruit valves (Supplementary Fig. 10). The live-cell imaging results suggested a progression of cell differentiation in valve epidermal cells from the base to the tip (Fig. 1a,b). We therefore compared the developmental trajectories of the valve tip epidermal cells during the shape transition between stages 13 and 14 (Fig. 2c,d). The epidermal cluster was identified as this cell type specifically expresses marker genes, such as *ATML1*, *FDH* and *DCR*^19–21^ (Supplementary Fig. 11 and Supplementary Data 2). Pseudotime analysis, whereby cells were ordered along a reconstructed differentiation trajectory using Monocle 2, showed that genes associated with the cell cycle (those encoding cyclins, cyclin-dependent kinases (CDKs) and proteins involved in cytokinesis) are expressed more in stage 13 than stage 14 samples (Fig. 2e–g, Extended Data Fig. 2 and Supplementary Data 2). Interestingly, comparison of the developmental trajectories of the epidermal cells revealed that both the expression level of cell cycle genes and the proportion of expressing cells were significantly reduced between stage 13 and stage 14, indicating a progressive decrease in cell division activity (Fig. 2e–g and Extended Data Fig. 2a–c).

**Extended Data Fig. 3:**
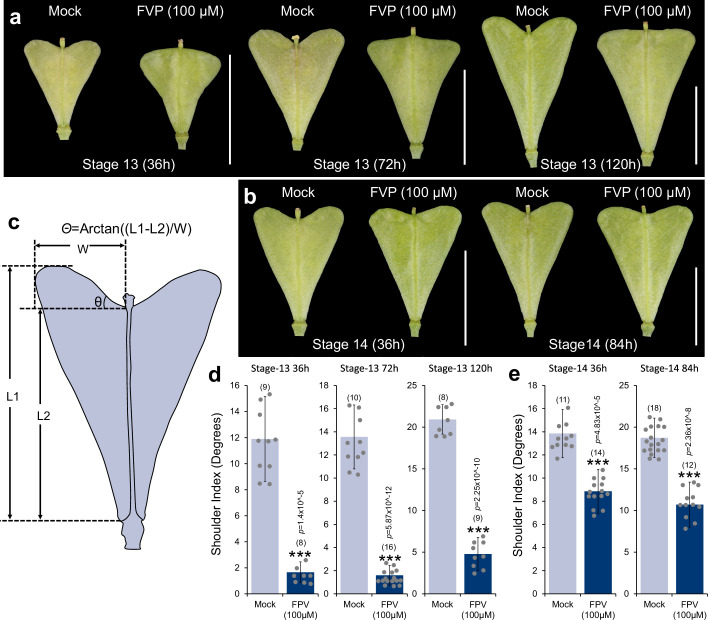
Effects of FVP treatment on *Capsella* fruit shape development. **a**, Time-course pictures of the fruits upon FVP and Mock treatment started from stage 13. Please note after 36 h, the stage-13 fruit has already developed to stage 14, 72 h to stage 15 and 120 h to stage 16. **b**, Time-course pictures of the fruits upon FVP and Mock treatment started from stage 14. Equivalent time points to stage 13 are shown. **c**, Schematic drawing to illustrate the shoulder index calculation. **d**, Shoulder index measurements of fruits from FVP and Mock treatment started from stage 13. **e**, Shoulder index measurements of fruits from FVP and Mock treatment started from stage 14. In **d** and **e**, values represent the mean of fruit shoulder index from the sample number indicated in the bracket, error bars represent SD. Error bars in **d** and **e** represent SD of at least 10 individual fruits. ***p < 0.001 (Single-sided Student’s t test). Scale bars, 5 mm in **a** and **b**.

To validate the scRNA-seq results, we generated GUS reporter lines (lines labelled with the β-glucuronidase (*GUS*) reporter gene), enabling us to track the expression of multiple cell cycle- and cytokinesis-associated genes, including *CrCDKB1;1*, *CrCYCB1;1*, *CrMAP65-3*, *CrMAP65-4*, *CrAUR2* and *CrPHGAP2*. We found these reporter lines to be expressed evenly in valves, with stronger expression detected in the valve tips during shape formation (Extended Data Fig. 2d). Live imaging results showed a relatively even distribution of cell division across the valve in stage 13, which slowed down by stage 14 (Fig. 1c,d). Together with the expression pattern of cell cycle reporters, these data suggest that sufficient levels of cell cycle and cytokinesis components are maintained to support cell proliferation throughout the valve, but with increased levels at the tips. Flavopiridol (FVP) is a CDK inhibitor that blocks cell cycle progression at the G1–S and G2–M phases^22^. In agreement with a role of cell division in reshaping the valve tips, FVP treatment substantially suppressed formation of the heart-shaped fruit by reducing valve tip growth (Extended Data Fig. 3a,b). These data suggest that maintenance of cell division is required to shape the apical part of the fruit. Consistent with this idea, fruits at stage 13 were more affected by FVP than those at stage 14 (Extended Data Fig. 3c–e).

Taken together, the scRNA-seq analysis provides transcriptomic support to the cellular growth data from the live-cell imaging analysis that cells in the valve maintain meristematic identity during the transformation in shape from gynoecium to the fertilized fruit. The stronger expression of the cell cycle genes specifically at the valve tips contrasts somewhat with the relatively shallow gradient of cell division measured at the epidermis by time-lapse experiments. It is possible that subepidermal layers retain stronger competency to divide at the tip, whereas epidermal divisions are more homogeneous.

**Fig. 3:**
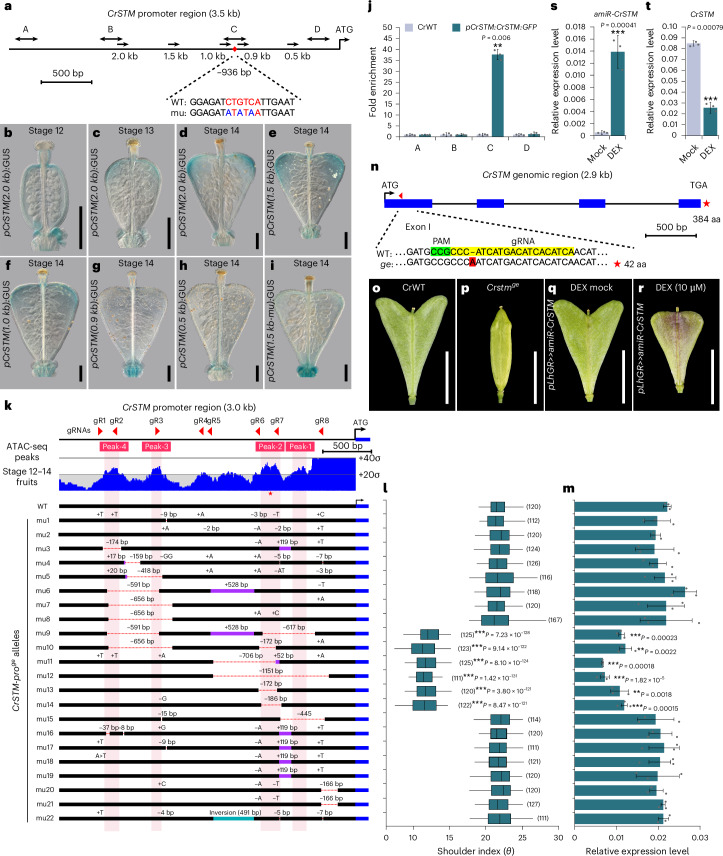
Functional analysis of the *CrSTM* locus reveals a positive feed-forward regulatory loop. **a**, Schematic of the 3.5 kb *CrSTM* promoter upstream of the start codon. The arrows indicate the promoter deletion series in the GUS reporter analyses in **b**–**i**. The double-headed arrows (A–D) indicate the regions used for ChIP analysis. The red diamond indicates the position of the STM-binding site (936 bp upstream of ATG start codon; CTGTCA red letters). The mutated version (mu; ATATAA) used in the 1.5 kb GUS reporter construct is aligned below. **b**–**d**, *CrSTM* expression patterns in *Capsella* fruits, shown by GUS staining at stage 12 (**b**), stage 13 (**c**) and stage 14 (**d**). **e**–**i**, *CrSTM* promoter activity, shown by GUS staining using 1.5 kb- (**e**), 1.0 kb- (**f**), 0.9 kb- (**g**), 0.5 kb- (**h**) and 1.5 kb-mutated (**i**) promoter:GUS reporter lines. **j**, ChIP analysis showing that the levels of CrSTM:GFP protein associated with regions A–D (defined by the double-headed arrows in panel **a**) of the *CrSTM* promoter were significantly higher in region C. **k**, ATAC-seq coverage (blue) on the 3.0 kb *CrSTM* promoter region (top panel) using stage 12–14 fruit samples. The grey lines mark the mean and standard deviation. The ATAC-seq peaks are numbered sequentially from ATG and shaded in light pink. The red triangles indicate the gRNAs used to generate the promoter-editing alleles and the red asterisk indicates the position of the STM-binding sequence. The bottom panel shows the 22 *CrSTM* promoter alleles. The deletions are indicated by dashed red lines and the insertions and inversions are shown by purple and green rectangles, respectively. **l**, Quantification of fruit shape by shoulder index (see also Extended Data Fig. 3c). The numbers of fruits used for quantification are indicated in parentheses. For each allele, at least three plants were sampled. Central lines indicates median values, the edges of the boxes define interquartile ranges, the whiskers represent 95% confidence intervals and the end points indicate minimum and maximum values. **m**, Analysis of *CrSTM* expression from stage 13/14 fruits of each allele. **n**, Schematic of the *CrSTM* genomic region (2.9 kb) with exons and introns indicated by blue rectangles and black lines, respectively. The single gRNA targeting the first exon is indicated by a red triangle. The genotyping result is presented below the graph with PAM sequence indicated in green, gRNA target sequence in yellow and inserted nucleotide in red. The red star represents the length in amino acids (42) of the truncated protein produced from the edited *CrSTM* gene. **o**,**p**, Fruit morphology of CrWT (**o**) and *Crstm*^*ge*^ (**p**) at stage 17. **q**,**r**, Fruit morphology of mock- (**q**) and DEX-treated (**r**) *pLhGR»amiR-CrSTM* lines at stage 17. **s**,**t**, Expression analyses of the *amiR-CrSTM* (**s**) and endogenous *CrSTM* transcripts (**t**) in stage 13/14 fruit samples upon DEX treatment. In **j**, **m**, **s** and **t**, the values represent the means of three biological replicates and the error bars represent s.d. Scale bars, 100 μm (**b**–**i**) and 5 mm (**o**–**r**). Statistical significance was determined by single-sided Student’s *t*-test (**P < 0.01; ***P < 0.001).

**Extended Data Fig. 4:**
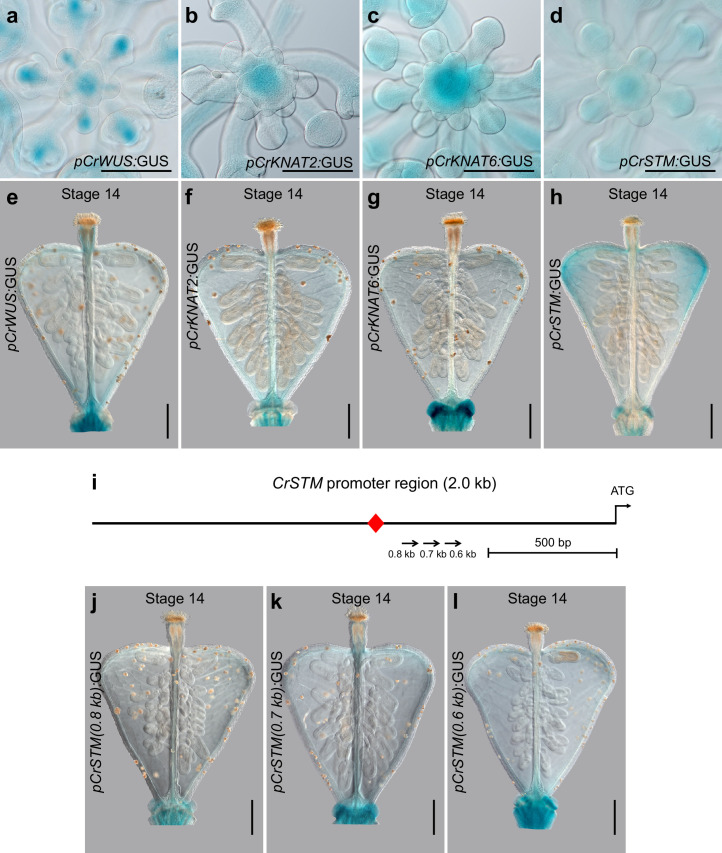
Expression analysis of candidate genes. **a**-**d**, Expression pattern of *CrWUS* (**a**), *CrKNAT2* (**b**), *CrKNAT6* (**c**) and *CrSTM* (**d**) shown by GUS staining with respective reporter lines in the shoot apical meristem (SAM), showing all genes are expressed in the SAM. **e**-**h**, Expression pattern of *CrWUS* (**e**) and *CrKNAT2* (**f**), *CrKNAT6* (**g**) and *CrSTM* (**h**) shown by GUS staining with respective reporter lines in the stage-14 fruits, showing only *CrSTM* (**h**) expression was detected in the fruit valve tips. **i**, Schematic of the 2.0 kb *CrSTM* promoter upstream of the start codon. The arrows indicate the promoter deletion series in the GUS reporter analysis. **j**-**l**, Expression pattern of *CrSTM(0.8* *kb)* (**j**), *CrSTM(0.7* *kb)* (**k**) and *CrSTM(0.6* *kb)* (**l**) shown by GUS staining with respective reporter lines in the stage-14 fruits, showing no *CrSTM* expression could be detected in the fruit valve tips. Scale bars, 100 μm.

Previously, we showed that valve tips have high auxin levels resulting from localized auxin biosynthesis^12^ coinciding with increased growth of tissue to create the heart shape^14^. This pattern of growth and auxin dynamics is analogous to the shoot apical meristem (SAM), where new organ primordia are initiated in a regular pattern through the formation of local auxin maxima and outgrowth^23,24^. In *Arabidopsis*, stem cell identity is maintained by an intricate network of key regulators, including genes encoding transcription factors, such as *WUSCHEL* (*WUS*), *SHOOTMERISTEMLESS* (*STM*), *KNAT1* (also known as *BREVIPEDICELLUS*), *KNAT2* and *KNAT6* (refs. ^5,25–27^). We speculated that specific expression of these meristem identity genes in the valve tips could be responsible for delaying differentiation in this region. To this end, we created GUS reporter lines for *Capsella* orthologues of these genes. Although they were all expressed in the SAM, only *CrSTM* revealed expression during fruit development (Extended Data Fig. 4a–h). Interestingly, a dynamic expression pattern of *CrSTM* coincided with progressive establishment of the heart-shaped fruit from stage 12 to stage 14 (Fig. 3a–d). In stage 14 fruits, *pCrSTM:GUS* expression was specifically localized in the valve tips (Fig. 3d), overlapping with the expression of auxin biosynthesis genes (*pCrYUC9:GUS* and *pCrTAA1:GUS*) and the *pDR5:GUS* auxin-signalling reporter^12^. Despite this expression pattern of the *CrSTM* promoter GUS reporter line, *CrSTM* transcripts were not detected in the scRNA-seq dataset. This may be due to a limitation of the scRNA technology in detecting low-abundance transcripts but with powerful ability to resolve the heterogenous cell types in a given organ, as was previously considered^28,29^. However, the specific signal observed in the reporter line suggested a potential role of *CrSTM* in regulating shape formation during *Capsella* fruit development and this was further investigated.

**Extended Data Fig. 5:**
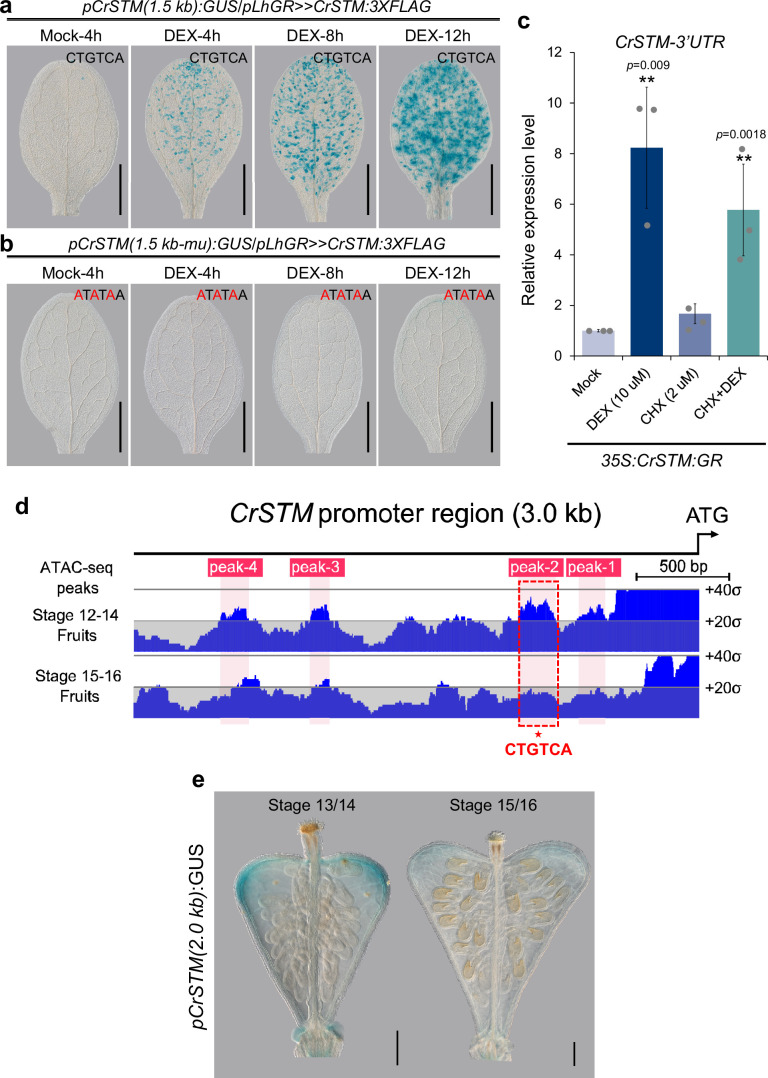
CrSTM directly activates its own expression through binding to an element in the *CrSTM* promoter. **a**, In the *pCrSTM (1.5* *kb):GUS/pLhGR»CrSTM:3×FLAG* lines, CrSTM:3*×*FLAG protein is over-expressed upon DEX treatment but not in the mock at the 4 hour time point. Over-expression of the CrSTM:3*×*FLAG protein, in turn, activates the *pCrSTM (1.5* *kb)* promoter shown by GUS staining in time-course records. Please note that the expression of GUS is accumulated over time with DEX treatment, suggesting a response of *pCrSTM (1.5* *kb)* promoter to the CrSTM:3×FLAG protein levels. **b**, In the *pCrSTM (1.5kb-mu):GUS/pLhGR»CrSTM:3×FLAG* lines, *pCrSTM (1.5kb-mu)* promoter does not respond to overexpression of CrSTM:3×FLAG proteins upon DEX treatment, suggesting that the STM-binding site is responsible for the activation of GUS by CrSTM:3xFLAG. **c**, In the 35S:CrSTM:GR lines, endogenous *CrSTM* expression (detected by probes targeting the 3’UTR) could be activated by CrSTM:GR proteins in the presence of DEX and the protein synthesis inhibitor cycloheximide (CHX), indicating that *CrSTM* is a direct target of CrSTM:GR. **d**, Assay for Transposase-Accessible Chromatin using Sequencing (ATAC-Seq) analysis of the *CrSTM* promoter region (~3.0 kb) in stage 12-14 fruits and stage 15-16 fruit samples. The grey lines mark the mean and standard deviation. ATAC-seq peaks are numbered sequentially from ATG and shaded in light pink. Please note that the chromatin region containing the STM-binding site (i.e., peak-2; the red-dashed line boxed region) becomes less accessible in stage 15-16 fruit compared with stage 9-14 fruits, while other regions, such as peak-3 and peak-4 remain unchanged. **e**, Expression of CrSTM in stage 13/14 and stage 15/16 fruits shown by GUS staining in a *pCrSTM(2.0* *kb):GUS* reporter line. Values in **c** represent the mean of three biological replicates and error bars represent SD. **, p < 0.01 (Single-sided Student’s t-test). Scale bars in **a** and **b**, 1 mm; **e**, 100 μm.

*CrSTM* encodes a class I KNOX transcription factor that controls plant growth and development by maintaining the stem cell population in the SAM^5,30^. Regulatory variations in the *STM* gene and its homologues are required to control axillary meristems and complex leaf shapes^31,32^. *STM* has also been implicated in the regulation of leaf shape diversity affecting patterns of growth proliferation and differentiation^33^. Next, we attempted to dissect the functional regions that facilitate the role of *CrSTM* in fruit shape determination. To this end, we generated a series of promoter deletions and narrowed the functional region required for valve tip expression of *CrSTM* down to a 93 base pair (bp) segment around 1,000 bp upstream from the transcription start site (Fig. 3a,e–h and Extended Data Fig. 4i–l). Within this region, we identified a hexamer, CTGTCA, that was previously reported as the recognition sequence for STM in *Arabidopsis*^34^. Intriguingly, this sequence is not present in the *AtSTM* promoter in the orthologous region (Supplementary Fig. 12) and mutating it in the *pCrSTM(1.5kb-mu):GUS* reporter line abolished expression in the valve tips (Fig. 3e,i). Using chromatin immunoprecipitation (ChIP), we found that CrSTM protein fused to green fluorescent protein (GFP) associated with a fragment containing the STM-binding site (Fig. 3j). In addition, using a dexamethasone (DEX)-inducible version of *CrSTM*, we could induce *pCrSTM(1.5k):GUS* expression and showed that this depends on the presence of the CTGTCA element (Extended Data Fig. 5a,b). Moreover, DEX-induced activation of *CrSTM* expression was unaffected by the presence of the protein synthesis inhibitor cycloheximide (CHX) (Extended Data Fig. 5c). These experiments show that *CrSTM* is autoregulated and sustains expression in the *Capsella* fruit through a positive feedback regulatory loop. This finding was furthermore supported by an assay for transposase-accessible chromatin with sequencing (ATAC-seq) experiment in which changes in chromatin accessibility in the region around the CTGTCA element were found to be associated with temporal *CrSTM* expression in the fruit (Fig. 3k and Extended Data Fig. 5d,e).

**Extended Data Fig. 6:**
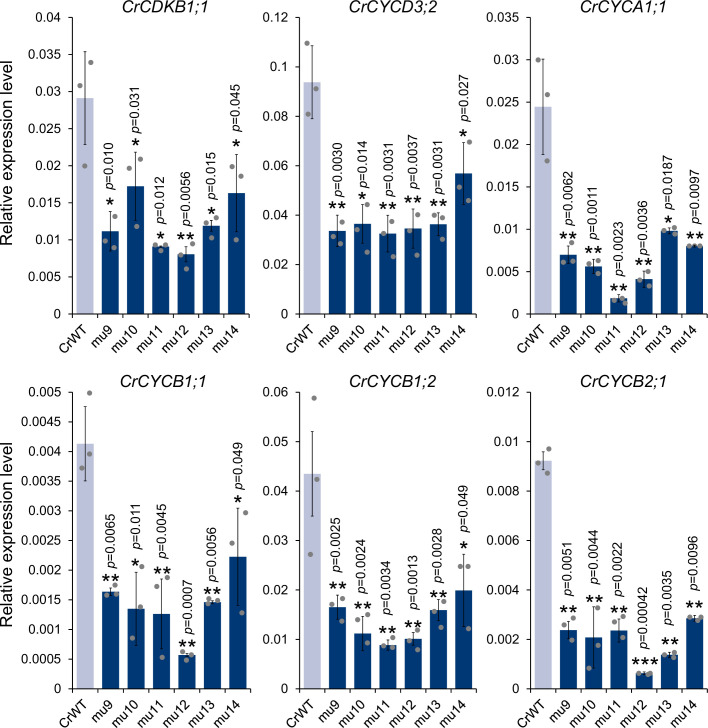
Expression analysis of cell cycle genes in the *CrSTM* promoter CRISPR lines. qRT-PCR analysis of gene expression in the stage 13/14 fruits of CrWT and each STM-binding site knock out line (*mu9*-*mu14*; details are provided in Fig. 3 in the main text). Values represent the mean of three biological replicates and error bars represent SD. *, p < 0.05; **, p < 0.01; ***, p < 0.001 (Single-sided Student’s t-test).

To further assess the functional importance of the STM-binding site in fruit shape determination compared with other regions of the *CrSTM* promoter, we created 22 gene-edited lines with diverse deletions in their *CrSTM* promoter region. To this end, we used a CRISPR–Cas9-based system with eight guide RNAs (gRNAs) distributed across a 3 kilobase (kb) region upstream of the *CrSTM* coding region (Fig. 3k and Supplementary Fig. 13). The effects of the deletions were evaluated by quantifying the fruit shape by shoulder index^12^ and *CrSTM* expression level (Fig. 3l,m). We found that mutant lines in which the STM-binding site was deleted (*mu9*–*mu14*) exhibited compromised *CrSTM* expression and reduced shoulder indices (Fig. 3k–m and Supplementary Fig. 14). Downregulation of *CrSTM* in these lines coincided with decreased expression of genes involved in cell cycle control (Extended Data Fig. 6). In these lines, however, heart shape formation was not completely abolished, suggesting that a basal level of *CrSTM* expression still has a role independent from *CrSTM* autoactivation in fruit shape determination. Altogether, these results further support the pivotal role of the STM-binding site in fruit shape determination, with little contribution from other regions of the *CrSTM* promoter.

The expression of *STM* and cell cycle reporters such as *CrCDKB1:1* and *CrCYCB1;1* towards the outer edge of the valve is largely complementary to the anisotropic growth area observed at medial regions of the *Capsella* fruit observed at stage 14 (Figs. 1h and 3d and Extended Data Fig. 2d). In addition to the medial region pushing the upper part of the valve outward, we suggest that higher proliferation and growth located at the edge of the shoulder may contribute to tip growth by pulling the cells located in the middle towards the shoulder, thereby making them grow more anisotropically.

**Extended Data Fig. 7:**
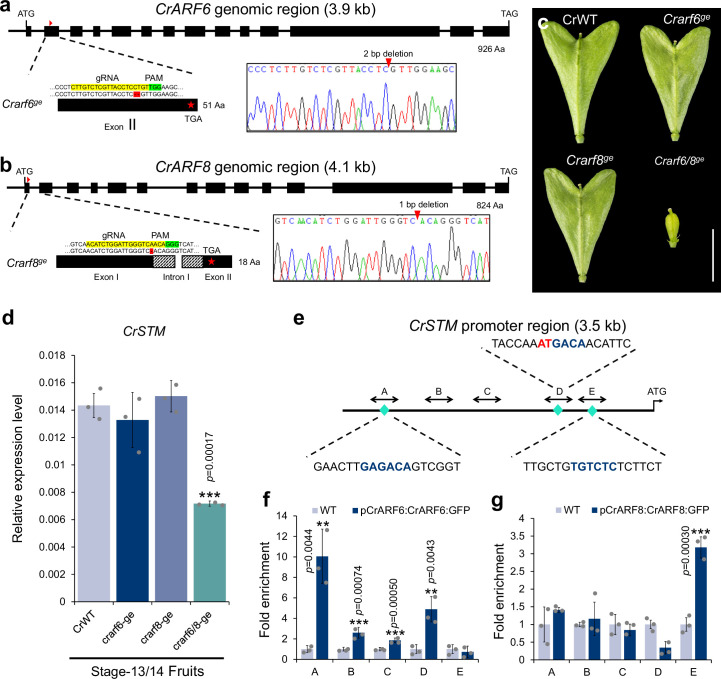
CrARF6/8 redundantly activate *CrSTM* expression in the fruit. **a**, Schematic showing the *CrARF6* gene structure. The gRNA targeting the second exon is indicated by a red triangle. The *Crarf6*^*ge*^ mutant was generated and confirmed by PCR-Sequencing of a 2-bp (CT) deletion in the second exon and the resultant protein was 51-aa in length compared with 926-aa in the WT. **b**, Schematic showing the *CrARF8* gene structure. The gRNA targeting the first exon is indicated by a red triangle. The *Crarf8*^*ge*^ mutant was generated and confirmed by PCR-Sequencing of a 1-bp (A) deletion in the first exon and the resultant protein was 18-aa in length compared with 824-aa in WT. The gRNA and PAM sequences are shaded in yellow and green, respectively. ATG and TAG indicate start and stop codons, respectively. **c**, Fruit morphology of the WT and *Crarf6*^*ge*^, *Crarf8*^*ge*^ and *Craf6/8*^*ge*^ mutants at stage 17. **d**, In the stage-13/14 fruits, expression of *CrSTM* remained unchanged in both the *Crarf6*^*ge*^ and *Crarf8*^*ge*^ single mutants, while its expression was significantly decreased in the *Crarf6/8*^*ge*^ double mutant, suggesting a redundant function of *CrARF6* and *CrARF8* in regulating *CrSTM*. **e**, Schematic showing the *CrSTM* promoter. The green diamonds indicate the auxin-responsive elements (blue letters). Double-headed arrows indicate the regions subjected to ChIP-qPCR analysis. **f** and **g**, Chromatin Immuno-Precipitation (ChIP) analysis of CrARF6:GFP (**f**) and CrARF8:GFP (**g**) associated with the *CrSTM* promoter. In **d**, **f** and **g**, values represent the mean of three biological replicates and error bars represent SD. **, p < 0.01; ***, p < 0.001 (Single-sided Student’s t-test). The scale bar in **c** represents 5 mm.

The autoregulatory expression of *CrSTM* raises the question of how it is initially kicked off. It was previously shown that *STM* in *Arabidopsis* is regulated partly by auxin response factors (ARFs) in the floral meristem^35^. Given that auxin dynamics at the valve tips plays a critical role in heart shape formation^12,14^, it is possible that *CrSTM* expression is affected by auxin. Two ARFs are particularly important for fruit growth in *Arabidopsis*—namely ARF6 and ARF8 (ref. ^36^)—and are therefore candidates for regulating *CrSTM* expression. We identified the closest orthologues of *ARF6* and *ARF8* in *Capsella* (*CrARF6* and *CrARF8*) and created single- and double-knockout mutants by gene editing (labelled with superscript ge) to assess their role in *Capsella* fruit development (Extended Data Fig. 7a,b). Although neither *Crarf6*^ge^ nor Cr*arf8*^ge^ single mutants have apparent defects in fruit valve development, the *Crarf6/8*^ge^ double mutant exhibited severe defects in valve growth after fertilization (Extended Data Fig. 7c). Interestingly, *CrSTM* expression was significantly reduced in the *Crarf6/8*^ge^ double mutant but not in either of the single mutants (Extended Data Fig. 7d) and ChIP experiments with GFP-tagged proteins showed that both CrARF6 and CrARF8 associate with regions of the *CrSTM* promoter (Extended Data Fig. 7e–g). These data suggest that auxin initiates *CrSTM* expression in the fruit through the redundant function of *CrARF6/8*. Once activated, *CrSTM* sustains its expression via autoregulation, thus extending the time window of cell proliferation and regulating the morphological change. The auxin-driven activation of *CrSTM* expression is different from the repressive effect on *STM* by ARFs, as previously reported in the *Arabidopsis* SAM to promote the initiation of flowers^35^, suggesting a context-dependent effect of auxin on *STM* expression.

**Extended Data Fig. 8:**
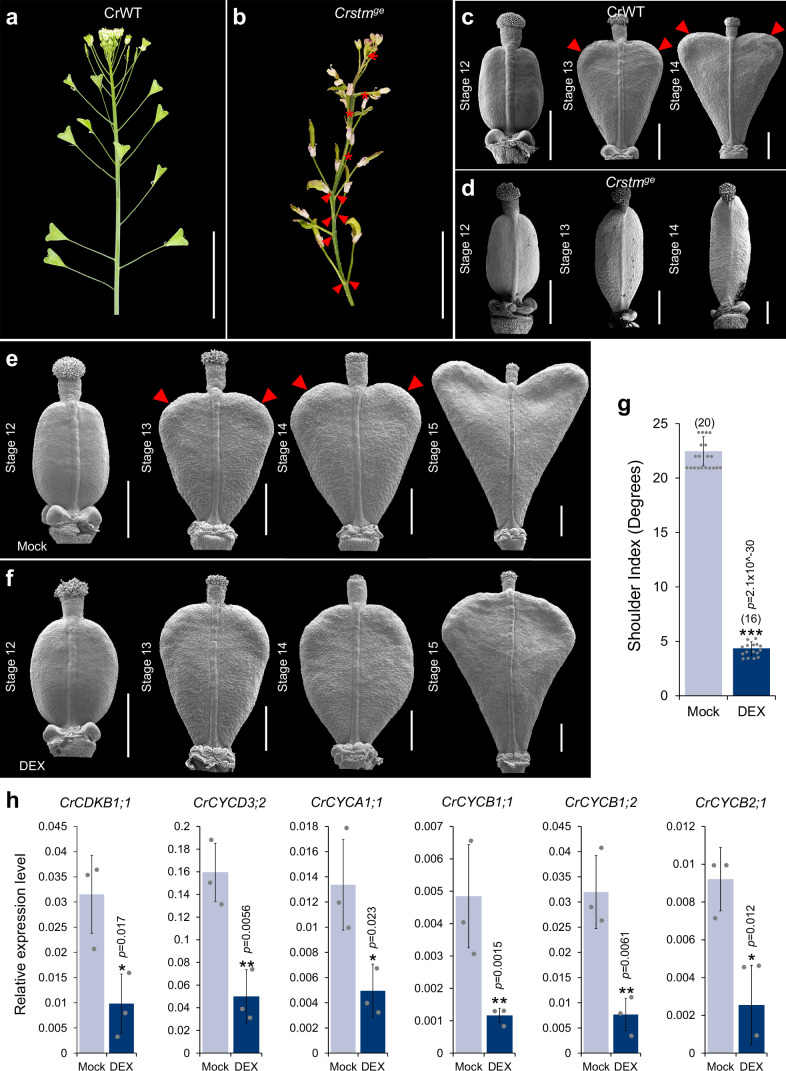
*Crstm*^*ge*^ mutation or expression knocking-down disrupts the fruit shape transition process. **a** and **b**, Inflorescence phenotype of the WT (**a**) and *Crstm*^*ge*^ mutant (**b**). Please note that *Crstm*^*ge*^ mutation generates pleiotropic defects in the inflorescence, including premature termination of the SAM (red arrow), fused pedicles (red asterisks) and altered phyllotaxis (red triangles). **c** and **d**, SEM analysis of WT (**c**) and *Crstm*^*ge*^ (**d**) fruits from stages 12 to 14. **e** and **f**, SEM analysis of Mock (**e**) and DEX-treated (**f**) fruits from stage 12 to 14 in *pLhGR»amiR-CrSTM* plants. **g**, Shoulder index measurements of fruits from DEX and Mock treated plants. **h**, qRT-PCR analysis of genes involved in the cell cycle showing that these genes are significantly down-regulated upon DEX treatment in stage 13/14 fruits. Error bars in **g** represent SD of at least 15 individual fruits and in **h** represent the SD of three biological replicates. Red triangles in **c** and **e** indicate the protrusions of the valve tips. In **g** and **h**, values represent the mean of three biological replicates and error bars represent SD. *, p < 0.05; **, p < 0.01; ***, p < 0.001 (Single-sided Student’s t-test). Scale bars, **a** and **b**, 3 mm; **c** to **f**, 500 μm.

To directly test the requirement for *STM* in fruit shape formation, we generated knockout lines of *CrSTM* using gene editing and targeting exon 1 of the *CrSTM* gene (Fig. 3n). Although many of these lines expectedly showed severe phenotypic defects from an early stage, as described for *stm* mutants in *Arabidopsis*^5^, some lines produced inflorescences and fruits (Extended Data Fig. 8a,b). Notably, these fruits completely lacked outgrowth of the valve tips and failed to undergo the characteristic change in shape from the disc-shaped gynoecium to the heart shape of fertilized fruits (Fig. 3o,p and Extended Data Fig. 8c,d). The fruits of the *Crstm*^ge^ mutant failed to set viable seeds, precluding us from further studying its contribution to fruit development. To overcome this, we generated a transgenic line expressing a DEX-inducible artificial microRNA (amiR) that targets the first exon of *CrSTM* (*pLhGR»amiR-CrSTM*). Upon DEX treatment, microRNA expression was induced and endogenous *CrSTM* mRNA levels were downregulated (Fig. 3s,t). Downregulation of *CrSTM* led to the production of smaller fruits lacking valve tips, thus abolishing the heart shape (Fig. 3q,r and Extended Data Fig. 8e–g). Compared with the strongly growth-impaired *Crarf6/8*^ge^ double-mutant fruits, the less dramatic effect seen in DEX-treated *pLhGR»amiR-CrSTM* fruits suggested that *CrARF6* and *CrARF68* promote fruit growth in *CrSTM*-independent ways, such as releasing the repressive effect of AP2 on cell growth, as described in *Arabidopsis*^36^. In fact, the phenotype of DEX-treated *pLhGR»amiR-CrSTM* transgenic fruits was very similar to that of FVP-treated fruits (Fig. 3r and Extended Data Fig. 3), suggesting that downregulation of *CrSTM* decreases cell division in the valve tips, which in turn perturbs the fruit shape development program in *Capsella*. In agreement with this, the expression of genes involved in cell cycle control was significantly reduced upon DEX treatment (Extended Data Fig. 8h). Together, these experiments demonstrate that the *Capsella STM* orthologue is required for the change in fruit shape from gynoecium to mature fruit.

**Extended Data Fig. 9:**
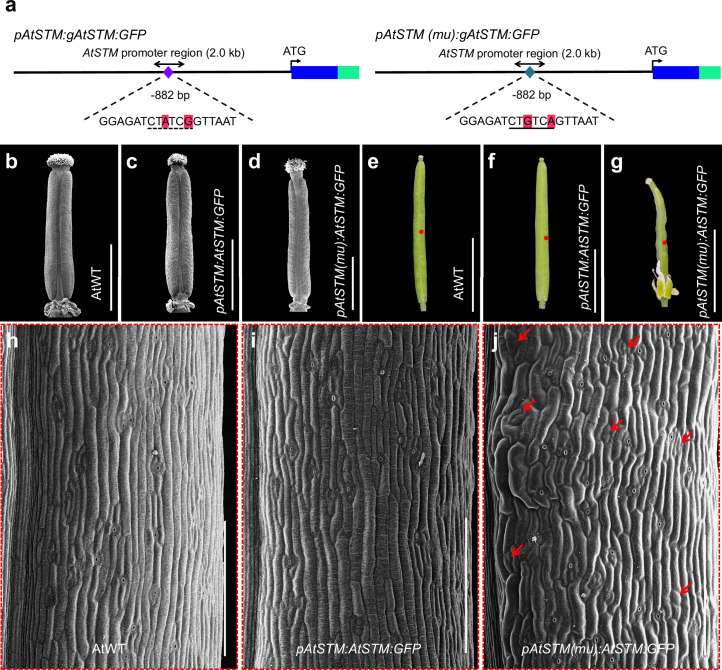
Gain-of-function mutation of the STM-binding site in *AtSTM* promoter disrupts fruit development. **a**, Schematic showing the *in situ* gain-of-function mutation of the STM-binding site in the *AtSTM* promoters. The different base-pairs are shaded in red; the STM-binding site is underlined with a solid line and the WT counterpart is underlined with a dashed line. Please note that in the *pAtSTM (mu):AtSTM:GFP* construct, the native CTATCG sequence is mutated into the CTGTCA STM-binding site, whereas the other promoter sequences remain unchanged. The length of each gene is not to scale. **b**-**d**, SEM images of the stage 11 gynoecia from AtWT (**b**), *pAtSTM:AtSTM:GFP* (**c**) and *pAtSTM (mu):AtSTM:GFP* (**d**) plants. **e**-**g**, Fruit morphology at stage 17 of AtWT (**e**), *pAtSTM:AtSTM:GFP* (**f**) and *pAtSTM (mu):AtSTM:GFP* (**g**) plants. **h**-**j**, SEM images of the epidermal cells from the fruits of AtWT (**h**), *pAtSTM:AtSTM:GFP* (**i**) and *pAtSTM(mu):AtSTM:GFP* (**j**), corresponding to the red dotted region shown in the respective genotype in **e** to **g**. The arrows in **j** indicate the cells are still ongoing cell division. Scale bars, **b** to **d**, 1 mm; **e** to **g**, 5 mm; **h** to **j**, 200 μm.

**Extended Data Fig. 10:**
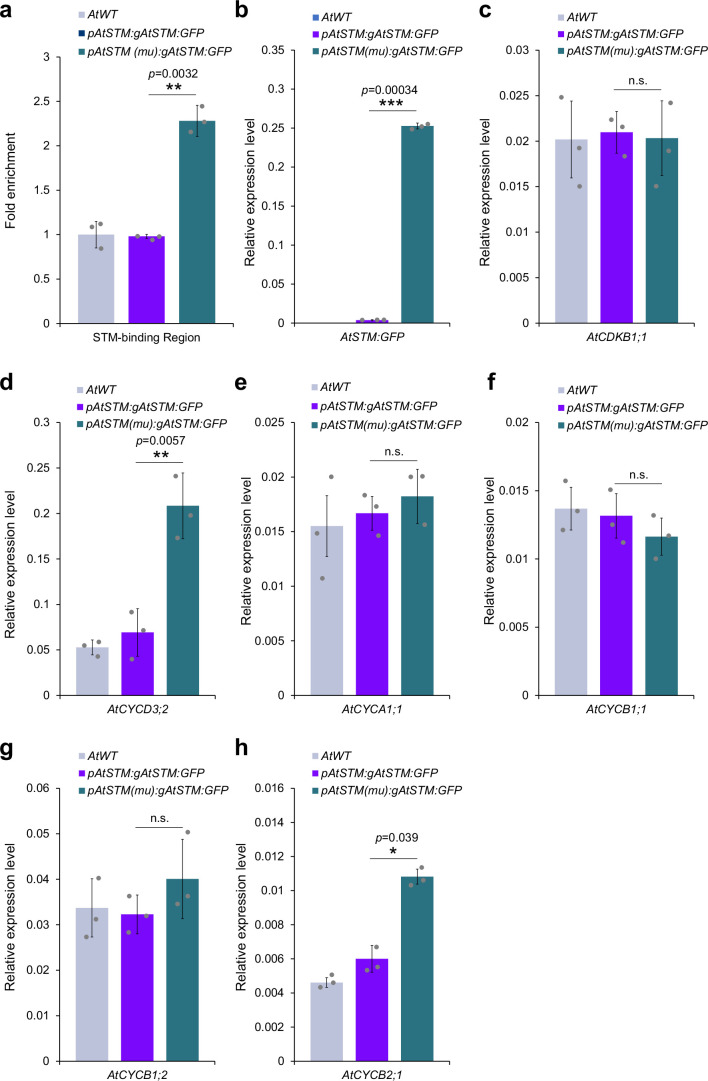
Expression analysis of cell cycle genes in the fruits of STM-binding site gain-of-function mutation in *Arabidopsis.* **a**, Chromatin Immuno-Precipitation (ChIP) analysis of AtSTM:GFP associated with the *AtSTM* promoter (shown in double arrows in Extended Data Fig. 9a). **b**-**h**, qRT-PCR expression analysis of *AtSTM:GFP* (**b**), *AtCDKB1;1* (**c**), *AtCYCD3;2* (**d**), *AtCYCA1;1* (**e**), *AtCYCB1;1* (**f**), *AtCYCB1;2* (**g**) and *AtCYCB2;1* (**h**) in the stage-13/14 fruits from AtWT, *pAtSTM:AtSTM:GFP* and *pAtSTM(mu):AtSTM:GFP* plants. Note that the expression levels of *AtCYCD3;2* (**d**) and *AtCYCB2;1* (**h**) are significantly up-regulated upon the ectopic expression of *AtSTM:GFP* in the fruits of *pAtSTM(mu):AtSTM:GFP* plants compared with those of *pAtSTM:AtSTM:GFP* plants. Values represent the mean of three biological replicates and error bars represent SD. n.s., not significant; *, p < 0.05; **, p < 0.01; ***, p < 0.001 (Single-sided Student’s t-test).

Since the STM-binding site is not present in the *AtSTM* promoter and *Arabidopsis* produces cylindrical fruits (Supplementary Fig. 12), we wanted to test the effect of introducing autoregulation of *AtSTM*. To this end, we engineered the *AtSTM* promoter to include the STM-binding site at the equivalent position in the promoter to that in the *CrSTM* gene (Extended Data Fig. 9a). A ChIP experiment showed that the AtSTM-GFP fusion protein indeed associates with this engineered region, coinciding with ectopic expression of *AtSTM* in the fruit (Extended Data Fig. 10a,b). Although gynoecia from transgenic *Arabidopsis* lines expressing this construct showed no morphological difference compared with the wild type, their fruits exhibited a shape change and overproliferation of epidermal cells (Extended Data Fig. 9b–j). Furthermore, such a phenotypic change was associated with upregulation of *AtCYCB2;1* and *AtCYCD3;2* expression (Extended Data Fig. 10c–h). Taken together, these data demonstrate that acquisition of the STM-binding element in the *AtSTM* promoter induces ectopic *STM* expression and cell proliferation leading to a morphogenetic change, showing that STM autoregulation is sufficient for fruit shape determination.

**Fig. 4:**
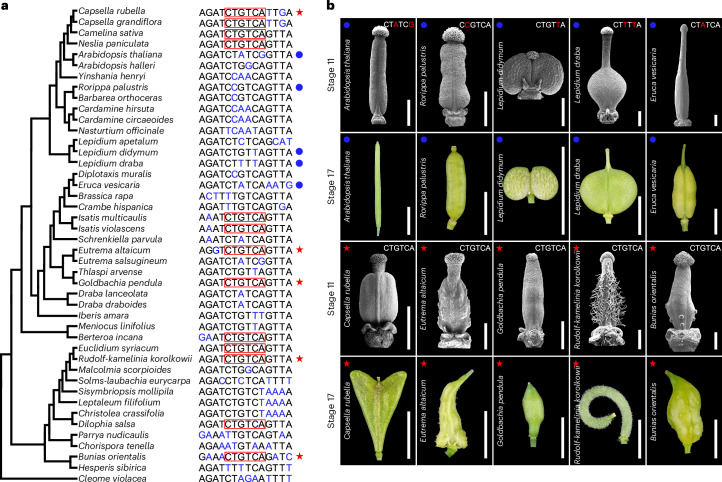
Evolution of the STM-binding sites associated with fruit shape diversification in Brassicaceae. **a**, Phylogenetic tree of 43 Brassicaceae species with *C. violacea* (Capparaceae) as the outgroup. The relationship between species was reconstructed based on published phylogenetic studies^35–37^. Regions orthologous to the STM-binding site in *C. rubella* are aligned. The red boxes indicate the consensus STM-binding site. Conserved sequences are shown in black and diverged sequences are shown in blue. The representative species with (red stars) and without (blue dots) the STM-binding site that were selected for morphological analysis are indicated to the right. **b**, SEM images of the gynoecia at stage 11 and whole-mount fruit images at stage 17 of Brassicaceae species with (red stars) or without (blue dots) the STM-binding site shown in **a**. Consensus or non-consensus STM-binding sites are shown in the SEM images with nucleotides that differ from consensus in red. Scale bars, 400 μm (SEM images) and 5 mm (whole-mount images).

The Brassicaceae family contains more than 3,800 species and is characterized by a diversity of fruit shapes that are derived from an ancestral cylindrical shape^4,37–40^. To test whether the STM-binding site exists in other *STM* orthologous genes, we analysed the promoter sequences of a panel of 43 Brassicaceae species with *Cleome violacea* (Cleomaceae) as the outgroup (Fig. 4a). Whereas the promoter of *C. violacea STM* (*CvSTM*) lacks the consensus sequence of the STM-binding site, this element appears to have evolved repeatedly within the Brassicaceae family (Fig. 4a). Remarkably, we noticed a perfect correlation between species harbouring the STM-binding site and those undergoing a shape change from the gynoecium to the mature fruit (Fig. 4b). We hypothesize that this could be due to post-fertilization cell divisions in the fruits, caused—at least in part—by *STM* expression and autoregulation. Moreover, in support of this proposal, our molecular evolution analysis revealed that the STM-binding site has been targeted by selection in Brassicaceae species with this element, but remains neutral in the counterpart region in species without this element (Supplementary Fig. 15). This analysis further substantiates the pivotal evolutionary significance of this particular 6 bp sequence in adaptation.

Nature is rich in morphological diversity, not least among fruits from flowering plants exhibiting an immense variation in shape. Our work shows that fruit morphogenesis depends on the maintenance of cell division and specific local patterns of growth. This process involves a positive and autoregulatory loop of the meristem identity factor STM, centred around the presence or absence of a STM-binding site in the promoter of the *STM* gene itself. The mechanism is initiated by local auxin-induced signalling and is required for the metamorphic shape change occurring between gynoecium and fruit formation. STM autoregulation relieves the morphological constraint imposed by the gynoecium shape. Since autoregulation of STM appears to have been co-opted independently multiple times within the Brassicaceae family, evolution of the regulatory STM-binding site may represent a particularly efficient mechanism for generating morphological diversity.

## Methods

### Plant materials and growth conditions

The *C. rubella* materials used in the study were in the *Cr22.5* ecotype background. The Brassicaceae species analysed in this study were ordered from the Germplasm Bank of Wild Species of China (Supplementary Table 1). The seeds were germinated on MS medium containing 1% sucrose, 0.8% agar and 10 µM gibberellin (G7645; Sigma–Aldrich) at 22 °C. The 10-day-old seedlings were then transplanted into soil in a controlled environment room (CER) at 22 °C under 16 h light/8 h dark conditions.

### Plasmid construction and plant transformation

To generate the cell membrane marker line, the *pUBQ10:acyl-YFP* plasmid^13^ was transformed into the *Cr22.5* ecotype background. To construct the *pCrSTM:GUS* (Carub.0002s0262; 1,966 bp), *pCrWUS:GUS* (Carub.0003s3208; 1,982 bp), *pCrKNAT2* (Carub.0002s1721; 4,400 bp), *pCrKNAT6* (Carub.0001s2296; 6,074 bp), *pCrCDKB1;1* (Carub.0005s2077; 447 bp), *pCrCYCB1;1* (Carub.0007s02791; 460 bp), *pCrMAP65-3* (Carub.0008s1164; 1,295 bp), *pCrMAP65-4* (Carub.0005s2760; 1,647 bp), *pCrAUR2* (Carub.0004s0570; 1,354 bp) and *pCrPHGAP2* (Carub.0006s1773; 3,220 bp) GUS reporter plasmids, each respective promoter was isolated via PCR using Phusion High Fidelity DNA Polymerase (M0530L; New England Biolabs) and inserted upstream of the *GUS* gene of the pCambia1301 vector. For *CrSTM* promoter activity analysis, a series of deletions were generated using the *pCrSTM:GUS* plasmid as a template. The sequences were inserted upstream of the *GUS* gene of each pCambia1301 vector. To generate the GFP-tagged transgenic lines, the full-length genomic fragment and promoter sequence (that is, *pCrSTM:CrSTM:GFP* (4,986 bp), *pCrARF6:CrARF6:GFP* (Carub.0001s2855; 7,157 bp), *pCrARF8:CrARF8:GFP* (Carub.0007s3229; 7,151 bp) or *pAtSTM(WT/mu):AtSTM:GFP* (AT1G62360; 4,490 bp)) were amplified and fused in-frame with the GFP coding sequence of the pCambia1302 vector. To construct the DEX-inducible *35S:CrSTM:GR* plasmid (the *CrSTM* coding region fused to the glucocorticoid receptor (GR)), the full-length *CrSTM* coding sequence was isolated from the fruit complementary DNA (cDNA) and inserted in-frame with GR in the pGTI0283 vector. To construct the two-component DEX-inducible *pLhGR»amiR-CrSTM* plasmid, artificial microRNA targeting the first exon of *CrSTM* was designed according to ref. ^41^. Briefly, the microRNA was synthesized as oligonucleotides and the functional sites in the microRNA319 backbone were substituted via series PCRs. The resultant amiR-CrSTM-miR319 PCR product was then integrated into the binary vectors using the Golden Gate cloning methods described in ref. ^42^. The *pLhGR»CrSTM:3×FLAG* plasmid was generated in a similar way to the *pLhGR»amiR-CrSTM* plasmid except that the *CrSTM* coding sequence was tagged with 3× FLAG. To construct the CRISPR–Cas9 genome editing plasmids, DNA sequences encoding gRNAs adjacent to PAM sequences (NGG) were designed using CRISPR-P 2.0 software^43^, which targets the first exon and eight sites that target the promoter of Carub.0002s0262. For gene editing of *CrARF6/8*, gRNAs targeting either exon 2 (*CrARF6*) or exon 1 (*CrARF8*) were designed. These gRNAs (Supplementary Table 2) were synthesized as oligonucleotides with Golden Gate cloning adaptors and were inserted downstream of *U6* promoters and then recombined with *pRPS5a:Cas9z:E9t* using Golden Gate cloning methods to produce the binary vectors. All vectors were verified by sequencing and introduced into *Agrobacterium tumefaciens* strain LBA4404 by cold-shock transformation. Details of the primers are provided in Supplementary Table 2.

Transformation of *Capsella* followed the floral dipping method optimized in ref. ^42^. The transformants were screened on MS plants with 1% sucrose and 0.8% agar containing either 40 mg l^−1^ hygromycin (10843555001; Roche) or 25 mg l^−1^ DL-phosphinothricin (P0159.0250; Duchefa). For each construct, at least ten independent transformants were obtained for further analysis. All of the analyses were conducted on the T2 generation of each representative transgenic line.

### Phenotyping and scanning electron microscopy

For whole-mount fruit phenotypes, stage 17 fruits of each genotype or Brassicaceae species were collected and photographed using a Nikon D850 camera equipped with a 105 mm prime lens. For scanning electron microscopy (SEM), the inflorescences of each genotype were fixed in a solution of formaldehyde, alcohol, and acetic acid and then dissected in 70% ethanol. The samples were dehydrated and critical point dried in CO_2_ followed by spotter coating with gold. The samples were examined using a Hitachi S-4800 scanning electron microscope with an acceleration voltage of 10.0 kV.

### Protoplast preparation and scRNA-seq

The protoplasts from the fruit valve tips were prepared via a previously described protocol with minor modifications^44^. Briefly, the tip regions of stage 13 and 14 fruit valves were dissected from 200–260 fruits with a knife under a light microscope and then digested in RNase-free enzymatic buffer (1.5% cellulase R-10, 0.4% macerozyme R-10, 0.6 M mannitol, 20 mM MES (pH 5.7), 20 mM KCl, 10 mM CaCl_2_ and 0.1% bovine serum albumin) for 2.5–3.0 h at 25 °C on a shaker at a speed of 40 r.p.m. The protoplasts were collected using cell strainers (40 μm in diameter; 352340; Falcon) and washed with W5 buffer (154 mM NaCl, 5 mM KCl, 125 Mm CaCl_2_ and 2 mM MES (pH 5.7)) two or three times to remove cell debris and incompletely digested tissues. The protoplasts were then concentrated with 0.6 M mannitol on ice, followed by a quality test (determined by trypan blue staining as >90% viable cells (as a percentage of total cells) for each sample). The concentration of protoplasts was finally adjusted to 1,000–1,200 cells per μl with 0.6 M mannitol. A total of 50,000 cells were prepared for each sample. The scRNA-seq experiments were performed with two independent biological replicates per stage.

To construct the sequencing library, the single-cell suspensions were loaded on a Chromium single-cell instrument (Chromium Controller; 10x Genomics) to generate single-cell gel beads in emulsion (GEMs). Then, the cDNAs were synthesized by reverse transcription on a PCR machine. The sequencing library was generated using the Chromium Single Cell 3′ GEM, Library & Gel Bead Kit v3 (1000075) and Chromium Single Cell B Chip Kit (1000073) (both 10x Genomics) according to the manufacturer’s instructions (Chromium Single Cell 3ʹ Reagent Kits v3 User Guide; CG000183 Rev A). The libraries were sequenced using an Illumina NovaSeq 6000 and two 150 bp paired-end kits. The raw scRNA-seq dataset comprised the following reads: read 1 (28 bp), read 2 (150 bp) and i7 index (8 bp).

### Bulk RNA-seq and correlation analysis

To identify the protoplasting-induced genes, total RNAs were isolated from fresh tissues and the protoplasts of valve tips using an SV Total RNA Isolation System. DNase I was then added to digest the genomic DNA (Z3100; Promega) following the manufacturer’s instructions. RNA-seq experiments were performed with three independent biological replicates. The RNA quality was determined using an Agilent 2100 Bioanalyzer. The sequencing libraries were constructed using a Hieff NGS Ultima Dual-mode mRNA Library Prep Kit for Illumina (12301ES96; YESEN) and sequenced on the Illumina NovaSeq 6000 according to the manufacturer’s instructions. The high-quality and clean reads generated by trimming the adaptors were mapped and annotated against the *C. rubella* v.1.0 genomic sequence^17^. EdgeR was used to identify the differentially expressed genes. In total, 65 differentially expressed genes were induced via the protoplasting process and used as controls for the effect of protoplasting in the scRNA-seq experiments.

Analysis of the correlation between scRNA-seq and bulk RNA-seq was conducted to determine whether the scRNA-seq data captured all of the biological processes in the valve tips. For the bulk RNA-seq datasets, we used the data matrix of the RNA-seq workflow on fresh valve tip samples. For the scRNA-seq datasets, we used the data from the Cell Ranger and Seurat workflows. Data integration and normalization were processed as previously described^18^. The function ‘cor.test’ in R was used to calculate the Spearman’s rank correlation between the bulk RNA-seq dataset and scRNA-seq dataset.

### Data integration, clustering and annotation

To align reads and generate gene–cell matrices, the raw dataset was processed using Cell Ranger 6.0.2 (10x Genomics) software with the default parameters. The nuclear genome (version 1.1) and the mitochondrial genome and associated GTF annotation files of *C. rubella* were downloaded from Phytozome (https://phytozome-next.jgi.doe.gov/) and the National Center for Biotechnology Information (https://www.ncbi.nlm.nih.gov/), respectively. These files were subsequently combined using the ‘cellranger mkref’ function to build the reference. Then, the gene–cell matrix was generated using the ‘cellranger count’ function. More than 90% of reads generated in all of the samples were tagged with cell barcodes by executing the ‘cellranger count’ function. From the two replicates, the one with more cells captured was subjected to in-depth sequencing (mean reads per cell > 40,000) and further analysis.

The resultant gene–cell matrix was then processed in the Seurat (version 4.3.0) package for in-depth data analysis, including quality control, normalization, dimension reduction, clustering and annotation. For quality control, we applied the following criteria: (1) cells with unique molecular identifiers numbering ~2,500–40,000 were selected for analysis; (2) the percentage of mitochondrial unique molecular identifiers was <5%; (3) cells containing fewer than 200 expressed genes were filtered out; (4) genes that were expressed in fewer than three cells were removed; and (5) genes with significant differential expression induced by the protoplasting process were excluded. To correct the variation caused by library preparation efficiency or sequencing depth, we used the ‘NormalizeData’ function to normalize the matrices. The top 2,000 HVGs were selected using the ‘FindVariableGenes‘ function following the variance-stabilizing transform method. For dimension reduction, the ‘RunPCA’ function was used to calculate 50 principal components of HVGs and the top 20 principal components representing an accumulating contribution rate of >85% were selected for downstream analysis. Next, we used the ‘FindNeighbors’ function on the top 20 principal components to compute the nearest neighbour networks. We then used the ‘FindClusters’ function to cluster cells with a ‘resolution of 0.7’ argument based on the Louvain method. The uniform manifold approximation and projection (UMAP) method was used to visualize the cell clusters. The cluster-specific or preferentially expressed genes were identified using the ‘FindAllMarkers’ function with the parameters ‘min.pct = 0.05, logfc.threshold = 0.5′. The ‘DotPlot’ and ‘VlnPlot’ functions were used to define the enrichments of marker genes in the corresponding cell clusters. An additional R package, DoubletFinder (version 2.0.2), was used to identify and remove the predicted doublet droplet from the clusters. The Plotly (version 4.10.1) package was used to generate a three-dimensional UMAP scatter graph. For data integration analysis, we merged the datasets from the two replicates using the Seurat (version 4.3.0) package workflow with the function merge’. The resultant merged dataset was processed by normalization, dimension reduction and clustering, then the ‘IntegrateLayers’ function with the parameter ‘method = CCAIntegration’ was used to perform integration. To visualize the two replicates and the merged dataset, we used the function ‘Dimplot’ with either the ‘group.by’ parameter or the ‘split.by’ parameter, respectively.

### Differentiation trajectory analysis

To reconstruct the developmental trajectory of the epidermal cells, cells expressing the specific marker genes (stage 13 clusters 4, 11 and 12 and stage 14 clusters 3, 12 and 14) were collected and grouped. To minimize the influence of stomata lineage on trajectory reconstruction, cells expressing *FAMA* were removed. To normalize the mRNA difference between cells, the ‘estimateSizeFactors’ and ‘estimateDispersions’ functions were applied. Subsequently, the monocle (version 2.18.0) package was used to infer the differentiation trajectory. A semi-supervised method was used to infer the genes involved in the corresponding biology process. The core developmental steps were calculated based on the DEGs from the initial HVGs using the ‘differentialGeneTest’ function (screening criteria: *q* value < 0.01). We used the DDRTree algorithm with the ‘reduceDimension’ function to reduce the dimensions. To align cells in the trajectory, the ‘orderCells’ function was performed; the root was established based on the maximum expression of *ATML1*, *FDH* and *DCR* in the trajectory. To visualize genes involved in the cell cycle and cytokinesis (Supplementary Data 2), we used the ‘plot genes in pseudotime’ function. The numbers of cells expressing these genes and expression values of corresponding genes were calculated from counts and visualized using the ggplot2 (version 3.4.2) package.

### Chemical treatment and phenotypic quantification

For application of the cyclin-dependent kinase inhibitor FVP (HY10006; MedChemExpress), 100 μM working solutions were prepared with water and Silwet (0.02%). The FVP and mock (water with 0.02% Silwet) solutions were dipped onto the stage 13 and stage 14 fruits under the microscope. The plants were then kept in the CER under long-day conditions (22 °C with 16 h light/8 h dark). The fruit phenotype was recorded at 36 and 48 h intervals. For DEX (D4902; Sigma–Aldrich) and CHX (01810; Sigma–Aldrich) treatment, 10 mM (DEX) and 2 mM (CHX) stock solutions were resolved in dimethyl sulfoxide (DMSO; D8418; Sigma–Aldrich). For the *35S:CrSTM:GR* lines, 10-day-old seedlings were transferred onto MS medium containing either 10 μM DEX, 2 μM CHX, 10 μM DEX plus 2 μM CHX or mock (with equal amount of DMSO) for 4 h on a shaker in the CER at a speed of 40 r.p.m., after which the samples were fixed in liquid nitrogen and subjected to gene expression analysis. For the *pLhGR»amiR-CrSTM* lines, 20 μM DEX solution with 0.02% Silwet or mock (water with 0.02% Silwet) solution was dipped onto the inflorescence (all flowering buds later than stage 12 on the inflorescences were removed using scissors) and kept under humid conditions for 24 h, then the plants were grown in the CER under long-day conditions (22 °C; 16 h light/8 h dark) to allow further development. Stage 13/14 fruits of mock- and DEX-treated fruits were fixed using liquid nitrogen and subjected to gene expression analysis. Fruit phenotypes were recorded at the respective developmental stages with a Nikon D850 camera or SEM. For the *pCrSTM(1.5* *kb WT/mu):GUS/pLhGR»CrSTM:3×FLAG* lines, 10-day-old seedlings were transferred into liquid MS medium containing either 10 μM DEX or mock (with equal amounts of DMSO) for 12 h on a shaker in the CER at a speed of 40 r.p.m. The samples were subjected to GUS staining at 4 h intervals.

The fruit shape was quantified as the shoulder index value, which was calculated with the anti-trigonometric function *θ* = Arctan((*L*_1_ – *L*_2_)/*W*) using the parameters described in ref. ^12^.

### Live-cell imaging and cell behaviour analysis

To conduct the live-cell imaging experiments, the pUBQ10:acyl-YFP plants were cultivated on soil in the CER under long-day conditions until they reached the bolting stage (22 °C; 16 h light/8 h dark). At stage 12, the fruits were dissected, transferred onto a slide and imaged, capturing yellow fluorescent protein signals at 24 h intervals using a Zeiss inverted-laser confocal microscope (Zeiss LSM 980) equipped with a water immersion objective (x25/0.95) and using ZEN Micropscopy Software. The excitation wavelength was set to 513 nm and the emission window was approximately 530 nm. Confocal stacks were acquired at a resolution of 1,024 × 1,024, with less than 0.5 μm in the *z* dimension. During the intervals between imaging sessions, the samples were maintained on Petri dishes containing 1/2 MS medium supplemented with vitamins (PM1011; Coolaber) and 1% sucrose in the CER under long-day conditions (22 °C; 16 h light/8 h dark). The acquired images were analysed using MorphoGraphX^45^. To quantify the cell area ratio, growth anisotropy and proliferation, the fluorescence was projected into a mesh, cell outlines were segmented and the relationships between cells were indicated across successive time points. Heatmaps illustrating the differences between two time points were generated, with the heatmap being shown on the fruit stage at the latter stage. Representative growth or proliferation maps were obtained from a single experiment. For quantification of cell growth and division, parameters were extracted at different time point using the MorphoGraphX software and a Wilcoxon test was used to test the significance.

### RNA extraction and expression analysis

The samples (fruits or seedlings) subjected to expression analysis were immediately fixed in liquid nitrogen. Total RNA was isolated from the samples using the SV Total RNA Isolation System, as mentioned above. 500 ng total RNA was reverse transcribed into cDNA in a 10 μl reaction with the SuperScript IV First-Strand Synthesis System (K1622; Thermo Fisher Scientific), according to the manufacturer’s instructions.

For real-time qPCR, gene-specific primers were designed (Supplementary Table 2) and verified by PCR followed by sequencing. The SYBR Premix Ex Taq (RR420DS; Takara Bio) was used to perform real-time qPCR with ROX as a reference dye on a StepOnePlus Real-Time PCR System (Life Technologies). The CT value of each gene was determined by normalizing the fluorescence threshold. The relative expression level of the target gene was determined using the ratio = 2^-ΔCT^ method. *CrUBQ10* was used as an internal control.

GUS histochemical assays were performed as previously described^12^.

### ChIP and ATAC-seq

For the ChIP experiment, stage 12–15 fruits from each genotype (GFP-tagged transgenic lines; see the section ‘Plasmid construction and plant transformation’) and wild-type plants were harvested and cross-linked in 1× phosphate-buffered saline plus 1% formaldehyde under vacuum for 10 min then 2 M glycine was added to a final concentration of 125 mM and vacuum was applied for another 5 min. Each sample containing 3.0 g of tissue was ground into a fine powder in liquid nitrogen and the nuclei were isolated with Honda buffer (0.44 M sucrose, 1.25% Ficoll, 2.5% Dextran T40, 20 mM HEPES KOH (pH 7.4), 0.5% Triton X-100, 10 mM MgCl_2_ and two tablets of Protease Inhibitor Cocktail (100 ml). Chromatin was released and fragmentated by sonication. After sonication, around one-twelfth (50 μl) of the DNA sample was taken out as input. The remaining DNA was subjected to anti-GFP immunoprecipitation using Pierce Protein G Magnetic Beads (88847; Thermo Fisher Scientific) coated with monoclonal anti-GFP antibody (11814460001; Roche) at 4 °C for 4 h. After immunoprecipitation, the beads were washed with the immunoprecipitation buffer and TE buffer then processed via the reverse crosslinking procedure in the presence of 10% sodium dodecyl sulfate at 65 °C for 12 h. The proteins in the DNA complex were digested using Proteinase K treatment at 45 °C for 1 h. After phenol/chloroform extraction, the DNA was visualized using GlycoBlue (AM9515; Thermo Fisher Scientific), precipitated in 70% ethanol, then dried and resolved in ChIP-grade water (W4502; Sigma–Aldrich). qPCR was performed using SYBR Premix Ex Taq on a StepOnePlus Real-Time PCR System (Life Technologies).

ATAC-seq experiments were performed following a previously described protocol^46^ with minor modifications. Briefly, ~0.5 g of stage 12–14 and stage 15–16 fruits were harvested and ground into fine power in liquid nitrogen. Approximately 50,000 nuclei were collected and washed using cold phosphate-buffered saline, then resuspended with cold lysis buffer (10 mM Tris-HCl (pH 7.4), 10 mM NaCl, 3 mM MgCl_2_ and 0.1% IGEPAL CA-630). The nuclei were then subjected to transposing reaction with Tn5 Transposase at 37 °C for 30 min and the DNA was purified using a Qiagen MinElute PCR Purification Kit (28006; Qiagen). The sequencing libraries were prepared by PCR amplification using NEBNext PCR master mix (M0541S; New England Biolabs). The amplified libraries were purified with AMPure beads (A63880; Beckman Coulter) and sequenced on an Illumina platform to generate 150-bp paired-end reads. For the ATAC-seq data analysis, the raw sequencing reads were first trimmed using fastp (version 0.20.0) to generate clean fastq files. After filtering out the low-quality reads (reads with a length below 35 bp and a base quality value of <10), the sequences were aligned to the *C. rubella* reference genome^6^ using Bowtie2 software^47^. The duplicated reads were marked and removed using sambamba (version 0.6.7) and bedtools (version 2.25.0)^48^. Peaks were then called using MACS2 (version 2.1.4)^49^ software with the screening criteria of a false detection rate of <0.05. Common peaks present in both replicates were identified and used for follow-up analysis. DeepTools (version 3.1.2) was used to map the density distribution of sequencing reads upstream and downstream of the transcription start site of each gene. For visualization, the datasets were converted to bigwig format using BamCoverage in DeepTools (version 3.1.2) with a bin size of 10 bp and then visualized using Integrative Genomics Viewer (version 2.4.14) software. The ATAC-seq experiments were performed with two independent biological replicates.

### Evolution analysis of the STM-binding sites

The promoter sequences of STM orthologues were isolated by TAIL-PCR (6108; Takara Bio) from the DNA of the representative Brassicaceae species (Supplementary Table 1). The 20-bp orthologous region containing the STM-binding site in the centre was extracted and aligned using MUSCLE version 3.8 (ref. ^50^) with default settings. The sample panel was divided into two groups: one containing the species with the STM-binding site (*n* = 21) and the other containing the species without the STM-binding site (*n* = 28). These two aligned files were separately inputted into the WebLogo online tool (https://weblogo.berkeley.edu/) to generate the sequence logo.

To evaluate selection on the flanking sequence on the STM-binding site, we computed the mean Shannon entropy for every 6 bp in a sliding-window mode for the 5′ and 3′ sequences (12 or 18 bp) flanking the STM-binding site and compared the values between groups with or without this site. For the control, we used the coding sequences of STM orthologous genes from Brassicaceae species with available genome sequences (https://github.com/mscharmann/tools/blob/master/extract_4fold_degenerate_sites.py). Next, we randomly sampled six sites from all of the fourfold sites, calculated the mean Shannon entropy value and repeated these two steps 1,000 times. Statistical comparisons among the three groups were performed using a two-sided Mann–Whitney *U* test in R (version 4.1.2).

### Quantification and statistical analysis

All of the statistical analyses were performed using Microsoft Excel. All of the measured data are presented as means ± s.d. (as specified in the figure captions) and sample sizes (*n*) are provided in the Methods and figure captions. Comparisons between groups for the qRT-PCR, fruit character and ChIP-qPCR analyses were performed in Microsoft Excel using single-sided Student’s *t*-tests and significance levels are marked with asterisks (*P < 0.05; **P < 0.01; ***P < 0.001).

### Statistics and reproducibility

For each GUS reporter construct, at least ten independent transformants were obtained for further analysis. All of the analyses were conducted on T2 generations of the representative transgenic lines. For phenotypic studies of transgenic lines using scanning electron microscopy or light imaging, specimens from at least five independent lines were analysed.

### Reporting summary

Further information on research design is available in the Nature Portfolio Reporting Summary linked to this article.

## Supplementary information

Supplementary Information Supplementary Figs. 1–15.

Reporting Summary

Supplementary Table 1 List of the Brassicaceae species used in this study.

Supplementary Table 2 List of the oligonucleotides used in this study.

Supplementary Data 1 scRNA-seq data quality and cluster-enriched genes.

Supplementary Data 2 Marker genes and cell cycle gene information.

## Data Availability

All of the data supporting the findings of this study are available within the paper and its Supplementary Information. The scRNA-seq data are available from the National Center for Biotechnology Information BioProject Database (www.ncbi.nlm.nih.gov) under the code PRJNA1067523.

## References

[1] Harding, M. J., McGraw, H. F. & Nechiporuk, A. The roles and regulation of multicellular rosette structures during morphogenesis. *Development* **141**, 2549–2558 (2014). 24961796 10.1242/dev.101444 PMC4067956

[2] Kidner, C. A. & Timmermans, M. C. P. Mixing and matching pathways in leaf polarity. *Curr. Opin. Plant Biol.* **10**, 13–20 (2007). 17140842 10.1016/j.pbi.2006.11.013

[3] Banda, J. et al. Lateral root formation in *Arabidopsis*: a well-ordered LRexit. *Trends Plant Sci.* **24**, 826–839 (2019). 31362861 10.1016/j.tplants.2019.06.015

[4] Eldridge, T. et al. Fruit shape diversity in the Brassicaceae is generated by varying patterns of anisotropy. *Development* **143**, 3394–3406 (2016). 27624834 10.1242/dev.135327 PMC5047655

[5] Long, J. A., Moan, E. I., Medford, I. & Barton, M. K. A member of the KNOTTED class of homeodomain proteins encoded by the *STM* gene of *Arabidopsis*. *Nature* **379**, 66–69 (1996). 8538741 10.1038/379066a0

[6] Wolters, H. & Jürgens, G. Survival of the flexible: hormonal growth control and adaptation in plant development. *Nat. Rev. Genet.* **10**, 305–317 (2009). 19360022 10.1038/nrg2558

[7] Willis, K. J. & McElwain, J. C. *The Evolution of Plants* 2nd edn (Oxford Univ. Press, 2014).

[8] Gottlieb, L. D. Genetics and morphological evolution in plants. *Am. Nat.* **123**, 681–709 (1984).

[9] De Vries, J. & Archibald, J. M. Plant evolution: landmarks on the path to terrestrial life. *New Phytol.* **17**, 1428–1434 (2018). 10.1111/nph.14975 29318635

[10] Beaulieu, J. M. & Donoghue, M. J. Fruit evolution and diversification in campanulid angiosperms. *Evolution* **67**, 3132–3144 (2013). 24151998 10.1111/evo.12180

[11] Stebbins, G. L. Adaptive radiation of reproductive characteristics in angiosperms, II: seeds and seedlings. *Annu. Rev. Ecol. Syst.* **2**, 237–260 (1971).

[12] Dong, Y. et al. Regulatory diversification of INDEHISCENT in the *Capsella* genus directs variation in fruit morphology. *Curr. Biol.* **29**, 1038–1046 (2019). 30827915 10.1016/j.cub.2019.01.057 PMC6428689

[13] Willis, L., Refahi, Y., Wightman, R. & Jönsson, H. Cell size and growth regulation in the *Arabidopsis thaliana* apical stem cell niche. *Proc. Natl Acad. Sci. USA* **113**, E8238–E8246 (2016). 27930326 10.1073/pnas.1616768113 PMC5187701

[14] Dong, Y. et al. HEARTBREAK controls post-translational modification of INDEHISCENT to regulate fruit morphology in *Capsella*. *Curr. Biol.* **30**, 3880–3888 (2020). 32795439 10.1016/j.cub.2020.07.055 PMC7544509

[15] Goméz-Felipe, A. et al. Two orthogonal differentiation gradients locally coordinate fruit morphogenesis. *Nat. Commun.* **15**, 2912 (2024). 38575617 10.1038/s41467-024-47325-1 PMC10995178

[16] Ripoll, J. J. et al. Growth dynamics of the *Arabidopsis* fruit is mediated by cell expansion. *Proc. Natl Acad. Sci. USA* **116**, 25333–25342 (2019). 31757847 10.1073/pnas.1914096116 PMC6911193

[17] Slotte, T. et al. The *Capsella rubella* genome and the genomic consequences of rapid mating system evolution. *Nat. Genet.* **45**, 831–835 (2013). 23749190 10.1038/ng.2669

[18] Zhang, T., Chen, Y. & Wang, J. W. A single-cell analysis of the *Arabidopsis* vegetative shoot apex. *Dev. Cell* **56**, 1056–1074 (2021). 33725481 10.1016/j.devcel.2021.02.021

[19] Takada, S., Takada, N. & Yoshida, A. *ATML1* promotes epidermal cell differentiation in *Arabidopsis* shoots. *Development* **140**, 1919–1923 (2013). 23515472 10.1242/dev.094417

[20] Yephremov, A. et al. Characterization of the *FIDDLEHEAD* gene of *Arabidopsis* reveals a link between adhesion response and cell differentiation in the epidermis. *Plant Cell* **11**, 2187–2201 (1999). 10559443 10.1105/tpc.11.11.2187 PMC144117

[21] Panikashvili, D., Shi, J. X., Schreiber, L. & Aharoni, A. The *Arabidopsis DCR* encoding a soluble BAHD acyltransferase is required for cutin polyester formation and seed hydration properties. *Plant Physiol.* **151**, 1773–1789 (2009). 19828672 10.1104/pp.109.143388 PMC2785978

[22] Carlson, B. A., Dubay, M. M., Sausville, E. A., Brizuela, L. & Worland, P. J. Flavopiridol induces G1 arrest with inhibition of cyclin-dependent kinase (CDK) 2 and CDK4 in human breast carcinoma cells. *Cancer Res.* **56**, 2973–2979 (1996). 8674031

[23] Galvan-Ampudia, C. S. et al. Temporal integration of auxin information for the regulation of patterning. *eLife* **9**, e55832 (2020). 32379043 10.7554/eLife.55832 PMC7205470

[24] Yang, W. et al. Molecular mechanism of cytokinin-activated cell division in *Arabidopsis*. *Science* **371**, 1350–1355 (2021). 33632892 10.1126/science.abe2305 PMC8166333

[25] Mayer, K. F. X. et al. Role of *WUSCHEL* in regulating stem cell fate in the *Arabidopsis* shoot meristem. *Cell* **95**, 805–815 (1998). 9865698 10.1016/s0092-8674(00)81703-1

[26] Venglat, S. P. et al. The homeobox gene *BREVIPEDICELLUS* is a key regulator of inflorescence architecture in *Arabidopsis*. *Proc. Natl Acad. Sci. USA* **99**, 4730–4735 (2002). 11917137 10.1073/pnas.072626099 PMC123716

[27] Ragni, L., Belles-Boix, E., Günl, M. & Pautot, V. Interaction of *KNAT6* and *KNAT2* with *BREVIPEDICELLUS* and *PENNYWISE* in *Arabidopsis* inflorescences. *Plant Cell* **20**, 888–900 (2008). 18390591 10.1105/tpc.108.058230 PMC2390738

[28] Yilmaz, S. & Singh, A. K. Single cell genome sequencing. *Curr. Opin. Biotechnol.* **23**, 437–443 (2012). 22154471 10.1016/j.copbio.2011.11.018 PMC3318999

[29] Baslan, T. & Hicks, J. Single cell sequencing approaches for complex biological systems. *Curr. Opin. Genet. Dev.* **26**, 59–65 (2014). 25016438 10.1016/j.gde.2014.06.004

[30] Endrizzi, K., Moussian, B., Haecker, A., Levin, J. Z. & Laux, T. The *SHOOT MERISTEMLESS* gene is required for maintenance of undifferentiated cells in *Arabidopsis* shoot and floral meristems and acts at a different regulatory level than the meristem genes *WUSCHEL* and *ZWILLE*. *Plant J.* **10**, 967–979 (1996). 9011081 10.1046/j.1365-313x.1996.10060967.x

[31] Zhang, C. et al. Spatiotemporal control of axillary meristem formation by interacting transcriptional regulators. *Development* **145**, dev158352 (2018). 30446629 10.1242/dev.158352 PMC6307885

[32] Uchida, N., Townsley, B., Chung, K.-H. & Sinha, N. Regulation of *SHOOT MERISTEMLESS* genes via an upstream-conserved noncoding sequence coordinates leaf development. *Proc. Natl Acad. Sci. USA* **104**, 15953–15958 (2007). 17898165 10.1073/pnas.0707577104 PMC2000400

[33] Kierzkowski, D. et al. A growth-based framework for leaf shape development and diversity. *Cell* **177**, 1405–1418 (2019). 31130379 10.1016/j.cell.2019.05.011 PMC6548024

[34] Spinelli, S. V., Martin, A. P., Viola, I. L., Gonzalez, D. H. & Palatnik, J. F. A mechanistic link between *STM* and *CUC1* during *Arabidopsis* development. *Plant Physiol.* **156**, 1894–1904 (2011). 21685178 10.1104/pp.111.177709 PMC3149926

[35] Chung, Y. et al. Auxin response factors promote organogenesis by chromatin-mediated repression of the pluripotency gene *SHOOTMERISTEMLESS*. *Nat. Commun.* **10**, 886 (2019). 30792395 10.1038/s41467-019-08861-3 PMC6385194

[36] Ripoll, J. J. et al. microRNA regulation of fruit growth. *Nat. Plants* **1**, 15036 (2015). 27247036 10.1038/nplants.2015.36

[37] Dong, Y. & Østergaard, L. Fruit development and diversification. *Curr. Biol.* **29**, R781–R785 (2019). 31430470 10.1016/j.cub.2019.07.010

[38] Huang, C. H. et al. Resolution of Brassicaceae phylogeny using nuclear genes uncovers nested radiations and supports convergent morphological evolution. *Mol. Biol. Evol.* **33**, 394–412 (2016). 26516094 10.1093/molbev/msv226 PMC4866547

[39] Couvreur, T. L. P. et al. Molecular phylogenetics, temporal diversification, and principles of evolution in the mustard family (Brassicaceae). *Mol. Biol. Evol.* **27**, 55–71 (2010). 19744998 10.1093/molbev/msp202

[40] Walden, N. et al. Nested whole-genome duplications coincide with diversification and high morphological disparity in Brassicaceae. *Nat. Commun.* **11**, 3795 (2020). 32732942 10.1038/s41467-020-17605-7 PMC7393125

[41] Schwab, R., Ossowski, S., Riester, M., Warthmann, N. & Weigel, D. Highly specific gene silencing by artificial microRNAs in *Arabidopsis*. *Plant Cell* **18**, 1121–1133 (2006). 16531494 10.1105/tpc.105.039834 PMC1456875

[42] Dong, Y., Hu, Z. C. & Østergaard, L. An optimized protocol to assess SUMOylation in the plant *Capsella rubella* using two-component DEX-inducible transformants. *STAR Protoc.* **3**, 101197 (2022). 35243380 10.1016/j.xpro.2022.101197 PMC8885766

[43] Lei, Y. et al. CRISPR-P: a web tool for synthetic single-guide RNA design of CRISPR-system in plants. *Mol. Plant* **7**, 1494–1496 (2014). 24719468 10.1093/mp/ssu044

[44] Wang, J. et al. An efficient and universal protoplast isolation protocol suitable for transient gene expression analysis and single-cell RNA sequencing. *Int. J. Mol. Sci.* **23**, 3419 (2022). 35408780 10.3390/ijms23073419 PMC8998730

[45] Strauss et al. Using positional information to provide context for biological image analysis with MorphoGraphX 2.0. *eLife* **11**, e72601 (2022). 35510843 10.7554/eLife.72601 PMC9159754

[46] Buenrosto, J. D., Wu, B., Chang, H. Y. & Greenleaf, W. J. ATAC-seq: a method for assaying chromatin accessibility genome-wide. *Curr. Protoc. Mol. Biol.* **109**, 21.29.1–21.29.9 (2015). 10.1002/0471142727.mb2129s109 PMC4374986 25559105

[47] Langmead, B. & Salzberg, S. L. Fast gapped-read alignment with Bowtie 2. *Nat. Methods* **9**, 357–359 (2012). 22388286 10.1038/nmeth.1923 PMC3322381

[48] Tarasov, A., Vilella, A. J., Cuppen, E., Nijman, I. J. & Prins, P. Sambamba: fast processing NGS alignment formats. *Bioinformatics* **31**, 2032–2034 (2015). 25697820 10.1093/bioinformatics/btv098 PMC4765878

[49] Zhang et al. Model-based analysis of ChIP-seq (MACS). *Genome Biol.* **9**, R137 (2008). 18798982 10.1186/gb-2008-9-9-r137 PMC2592715

[50] Edgar, R. C. MUSCLE: multiple sequence alignment with high accuracy and high throughput. *Nucleic Acids Res.* **32**, 1792–1797 (2004). 15034147 10.1093/nar/gkh340 PMC390337

