# Supplementary material for: Evolution of a SHOOTMERISTEMLESS transcription factor binding site promotes fruit shape determination
Source: Nat Plants. 2024 Dec 12;11(1):23–35. doi: 10.1038/s41477-024-01854-1 (PMC11757149; doi:10.1038/s41477-024-01854-1)
Supplement: Supplementary file 1 — Supplementary Figs. 1–15. [file 41477_2024_1854_MOESM1_ESM.pdf]

nature plants

Supplementary information https://doi.org/10.1038/s41477-024-01854-1

Evolution of a SHOOTMERISTEMLESS transcription factor binding site promotes fruit shape determination

In the format provided by the authors and unedited

Title: Evolution of a SHOOTMERISTEMLESS transcription factor binding site promotes fruit-shape determination

Authors: Zhi-Cheng Hu, Mateusz Majda, Hao-Ran Sun, Yao Zhang, Yi-Ning Ding, Quan Yuan, Tong-Bing Su, Tian-Feng Lü, Feng Gao, Gui-Xia Xu, Richard S. Smith, Lars Østergaard* and Yang Dong*

Supplementary Fig. 1-Fig. 15

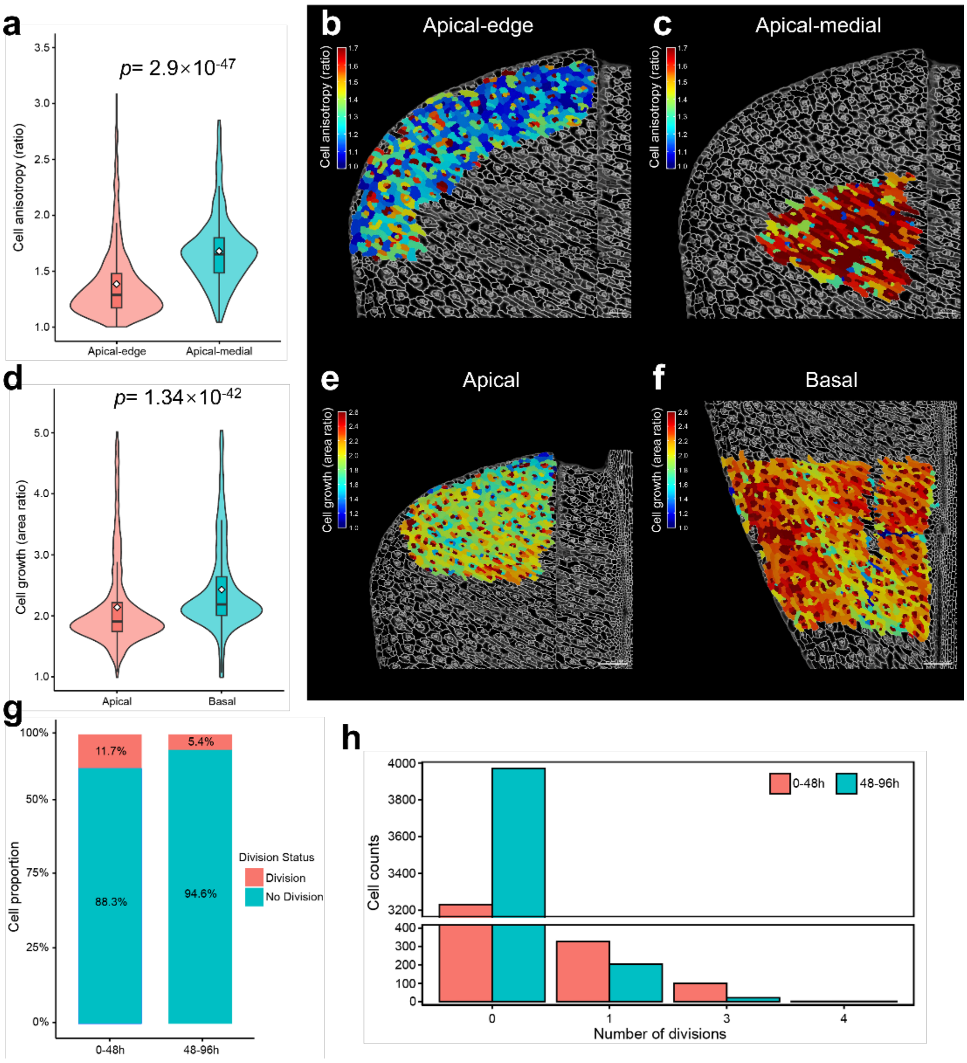

Supplementary Fig. 1 Analysis of cell growth and division in the *Capsella* fruits

a, Violin plot of anisotropy measurements in the apical-edge (531 cells) and apical-medial (337 cells) regions shows mean anisotropy values of 1.388 for the top and 1.682 for the bottom. A Wilcoxon test revealed a highly significant difference (p = 2.90 x 10^-47). **b** and **c**, Heatmaps illustrate anisotropy ratios in the apical-edge (**b**) and apical-medial (**c**) regions, ranging from 1 in blue (no anisotropy) to 1.7 in red (high anisotropy). **d**, Violin plot comparing growth ratios between the apical (710 cells) and basal (1216 cells) regions, showing significant differences (p = 1.34 x 10^-42). **e** and **f**, Heatmaps display growth ratios from 1 in blue (no change) to 2.6 in red (high growth) in the apical (**e**) and basal (**f**) regions. **g**, Bar plot showing the proportion of dividing vs. non-dividing cells during 0-48h and 48-96h, with 11.7% of cells dividing during 0-48h and 5.4% during 48-96h. **h**, Bar plot showing the number of cell divisions across 0-48h and 48-96h, where most cells underwent zero divisions, followed by smaller numbers undergoing one or more divisions. The y-axis includes breaks to visualize differences in cell counts between division categories. In box plots of **a** and **d**, the central line indicates the median, the edges of the box define the interquartile range, and the whiskers extend to the most extreme values. Scale bars, **b**, **c** and **e**, **f**, 50 µm.

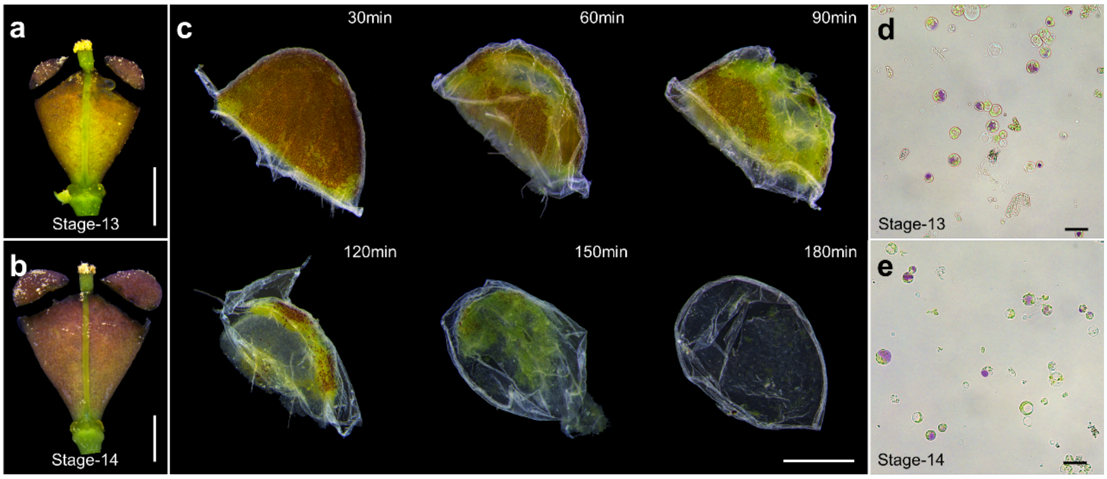

Supplementary Fig. 2 Sample preparation for the scRNA-seq analysis

a and **b**, Sampling stages. The valve tips of stage-13 (**a**) and stage-14 (**b**) fruit were harvested for protoplast preparation. **c**, Time-course record of the enzymatic digestion process of the stage-14 valve tips. Please note that the digestion was nearly complete after 150 min and fully complete after 180 min. **d** and **e**, The final protoplasts used for scRNA-seq analysis. The protoplasts from stage 13 (**d**) and stage 14 (**e**) fruit were shown. Scale bars, **a** and **b**, 300 μm; **c**, 150 μm; **d** and **e**, 50 μm.

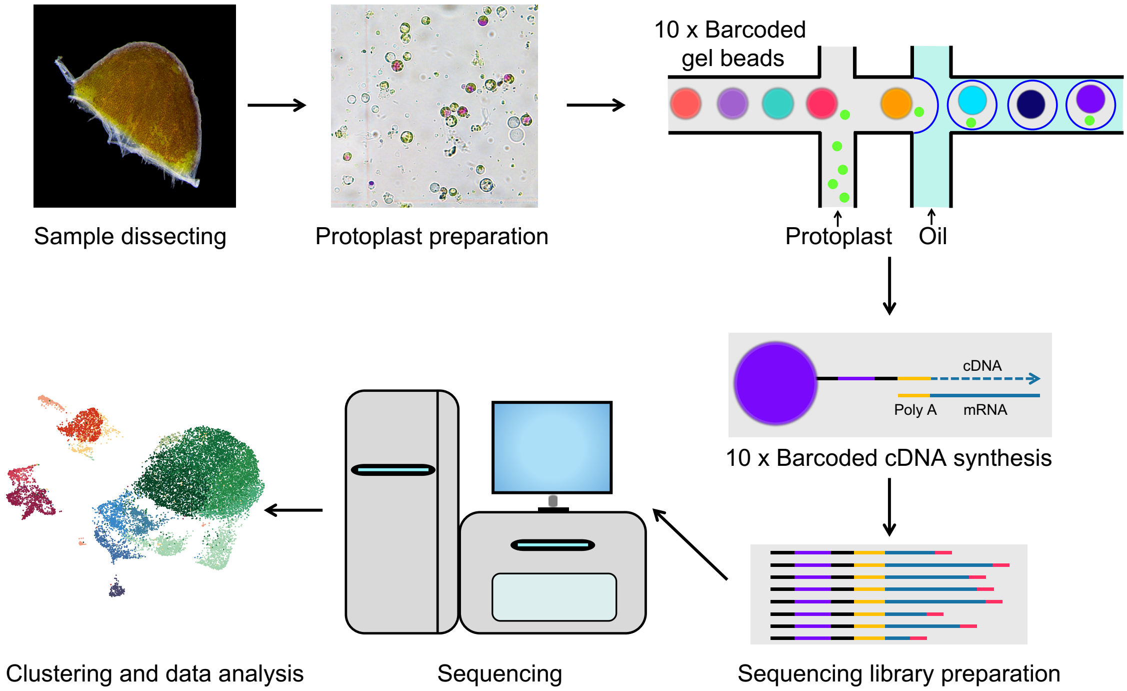

Supplementary Fig. 3 Workflow of the scRNA-seq analysis process

The fruit valve tips were dissected from fruit and digested to protoplast using RNase-free enzymatic buffer. The single-cell suspensions were loaded on a 10x platform to generate single-cell GEMs. After library preparation and sequencing, raw data was counted using Cell Ranger software to get a gene-cell matrix, and the Seurat package was used to visualize a UMAP plot for sample scRNA-seq transcriptome.

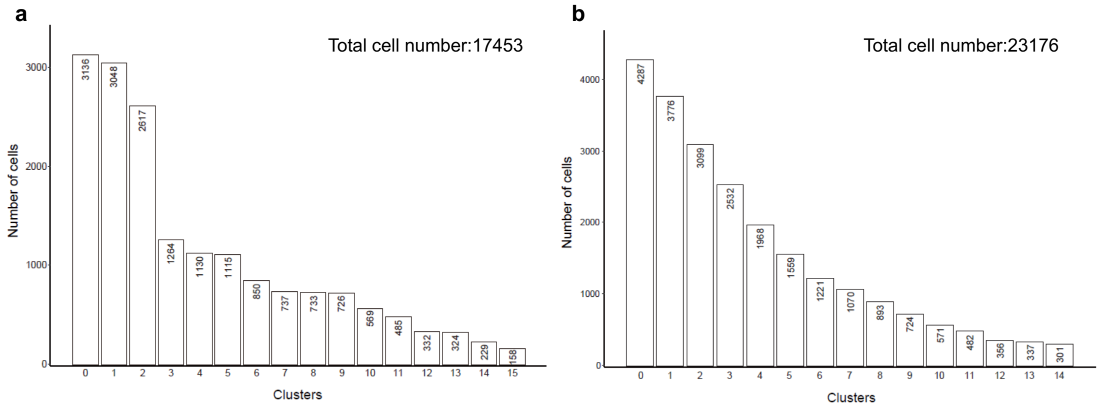

Supplementary Fig. 4 Cell numbers in each cluster of the scRNA-seq analysis

a and **b**, The cell numbers in each cluster from stage 13 fruit samples (**a**) and stage 14 fruit samples (**b**).

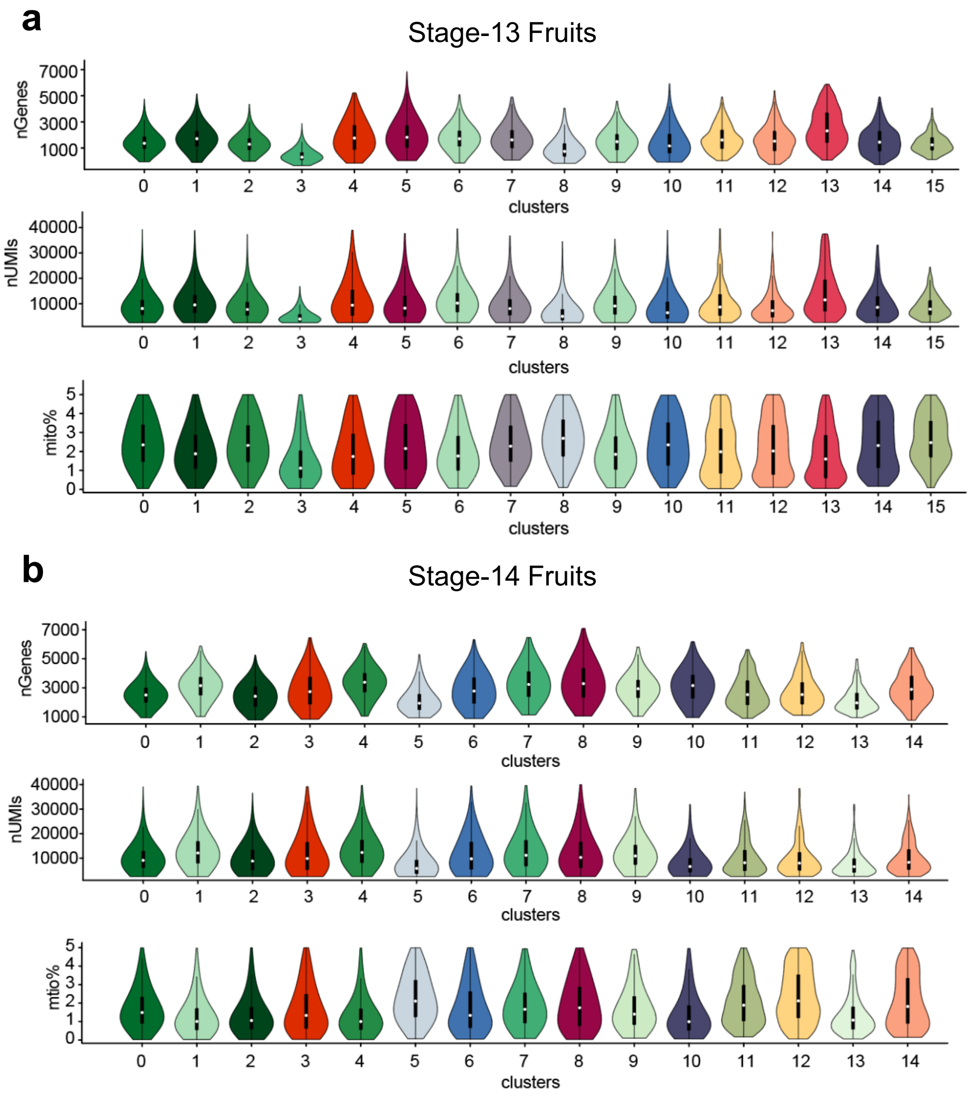

Supplementary Fig. 5 scRNA-seq data quality parameters

The data quality details of each cell cluster in the datasets of Stage 13 (**a**) and Stage 14 (**b**) produced from the workflow provided in the method section. nGene indicates the number of unique genes, nUMIs indicates the number of unique molecular identifiers and %mito indicates the percentage of mitochondrial gens expressed in the total scRNA-seq transcriptome of each cluster, respectively.

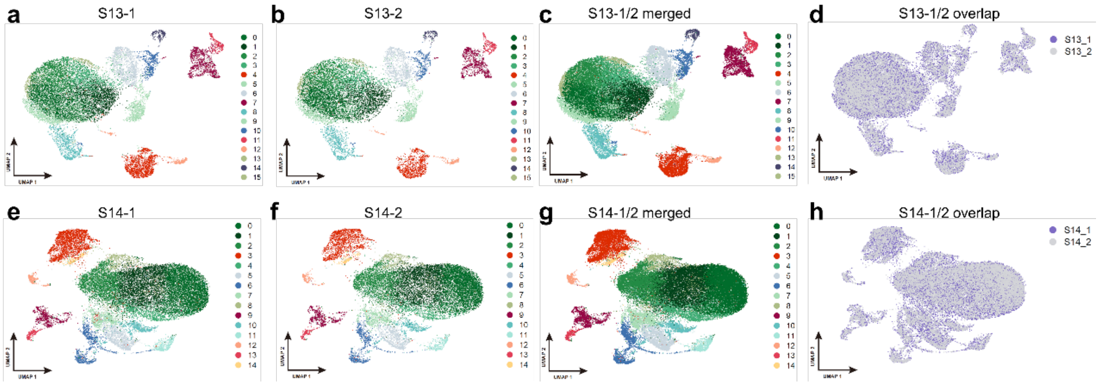

Supplementary Fig. 6 Reproducibility test of scRNA-seq data

a, UMAP projection of scRNA-seq datasets replicate 1 of stage 13 (S13-1) fruit sample. **b**, UMAP projection of scRNA-seq datasets replicate 2 of stage 13 (S13-2) fruit sample. **c**, UMAP projection of scRNA-seq datasets combined by replicate 1 and replicate 2 from stage 13 fruit sample. **d**, Cluster overlap between S13-1 (purple) and S13-2 (grey) dataset. **e**, UMAP projection of scRNA-seq datasets replicate 1 of stage 14 (S14) fruit sample. **f**, UMAP projection of scRNA-seq datasets replicate 2 of stage 14 fruit sample. **g**, UMAP projection of scRNA-seq datasets combined by replicate 1 and replicate 2 from stage 14 fruit sample. **h**, Cluster overlap between S14-1 (purple) and S14-2 (grey) dataset. Please note that the consistent cell cluster overlap between the two replicates on the UMAP projection, indicating high reproducibility of the experiment. Cells and clusters are annotated as shown in Fig. 2**a** and 2**b**.

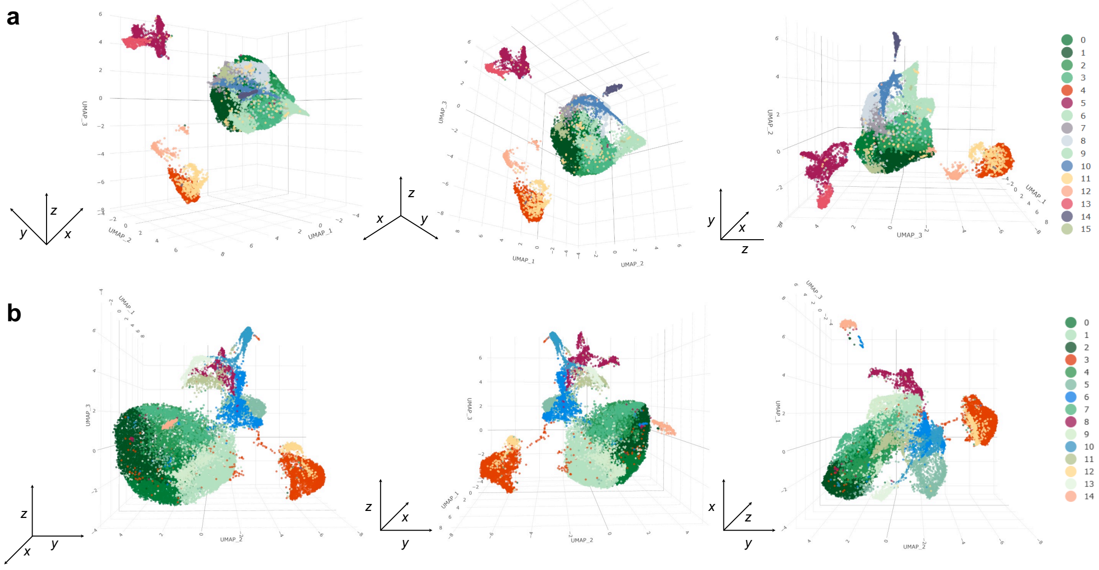

Supplementary Fig. 7 3D views of the scRNA-seq transcriptomic cell clusters

a, Visualization of cell clusters by 3D UMAP scatterplots of stage 13 fruit samples. **b**, Visualization of cell clusters by 3D UMAP scatterplots of stage 14 fruit samples. Cluster name and colors are the same as in Fig. 2**a** and 2**b**.

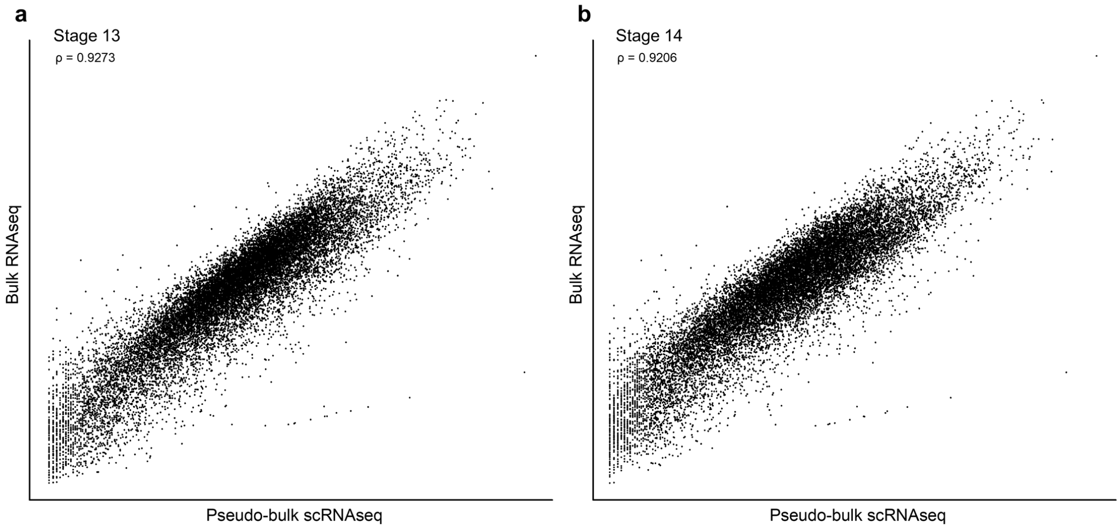

Supplementary Fig. 8 Correlation analysis of scRNA-seq and bulk RNA-seq data

a, The correlation index of fresh tissue bulk RNA-seq data and pseudo-bulk scRNA-seq data of stage 13 fruit sample. **b**, The correlation index of fresh tissue bulk RNA-seq data and pseudo-bulk scRNA-seq data of stage 14 fruit sample. The indexes are calculated by Spearman's rank correlation method.

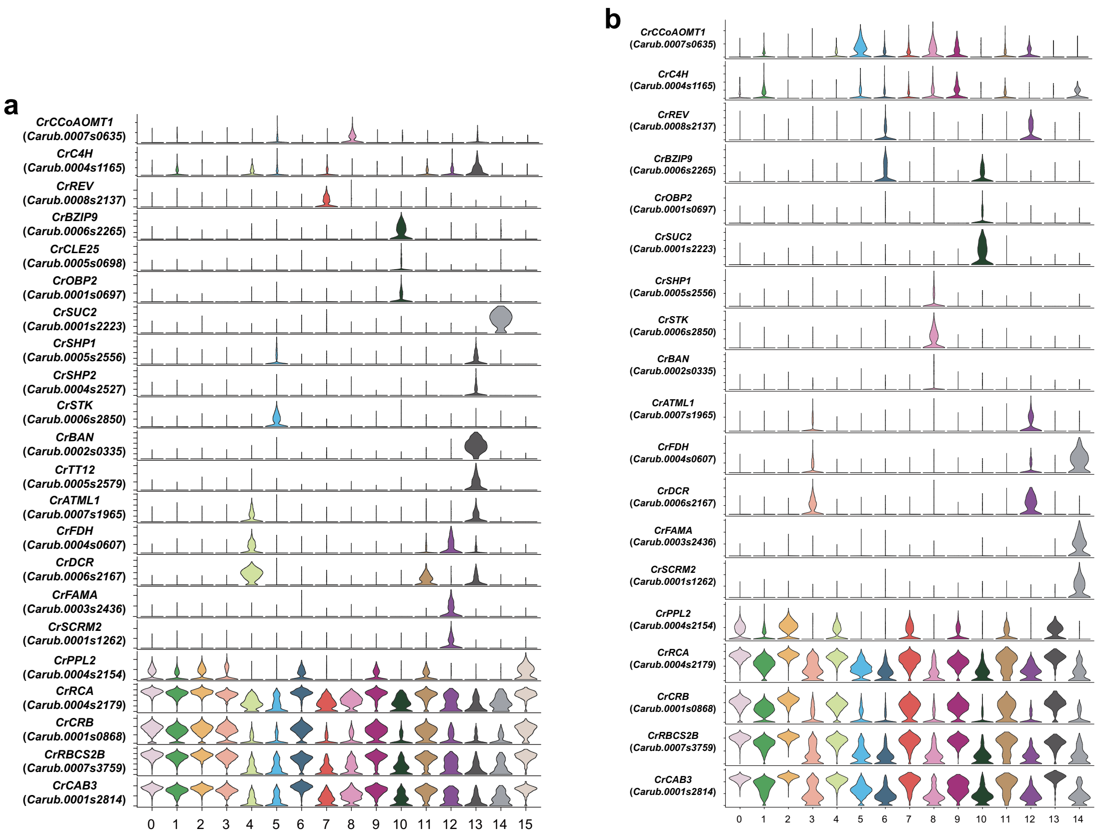

Supplementary Fig. 9 Expression patterns of representative cluster-specific makers genes on UMAP

a, Expression patterns of markers genes in stage 13 cell altas by Violin plot. **b**, Expression patterns of markers genes in stage 14 cell altas by Violin plot. *CrCCoAOMT1* and *CrC4H* are used to identify endocarp cell clusters. Vascular cell clusters are classified by the expression of *CrREV*, *CrBZIP9*, *CrCLE25*, *CrOBP2* and *CrSUC2*. Embryo cell clusters are identified by expressing transcript of *CrSHP1*, *CrSHP2*, *CrSTK*, *CrBAN* and *CrTT12*. *CrATML1*, *CrFDH* and *CrDCR* are related to the epidermis cell clusters. *CrFAMA* and *CrSCRM2* are specifically expressed in guard cell clusters. Mesophyll cell clusters were identified by photosynthetic related genes, such as *CrPPL2*, *CrRCA*, *CrCRB*, *CrRBCS2B* and *CrCAB3*. The full name of selected genes and associated references are provided in Supplementary Sheet 2.

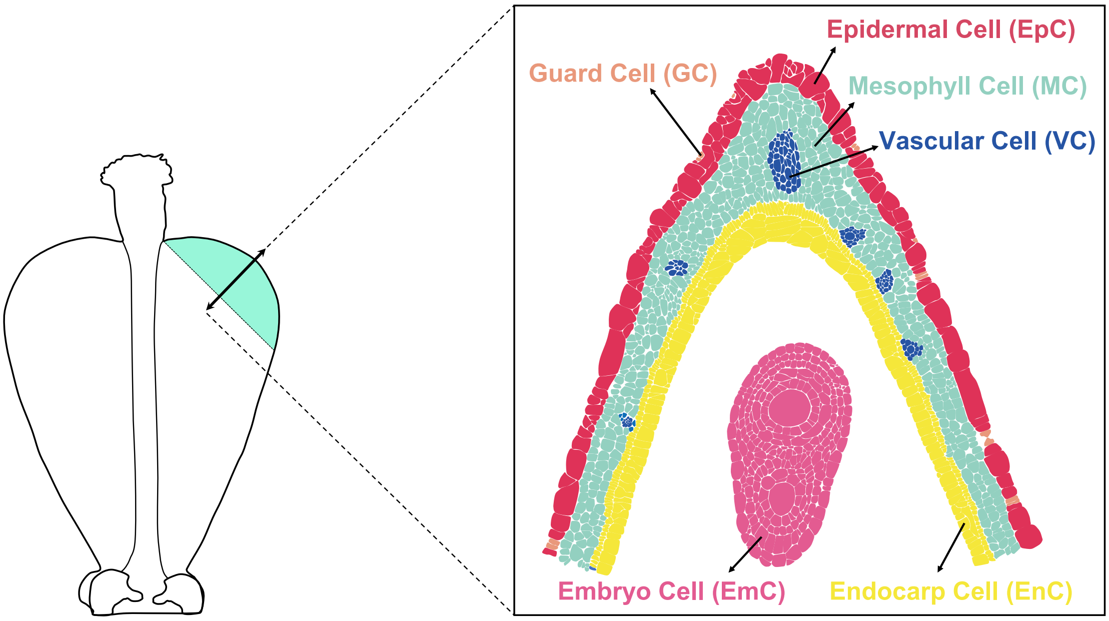

Supplementary Fig. 10 Schematic of anatomy and cell types of the valve tips

The double-head arrow indicates the direction of the section from a stage-13 fruit. Note that different cell types or tissues were colored based on the section in a 1:1 ratio.

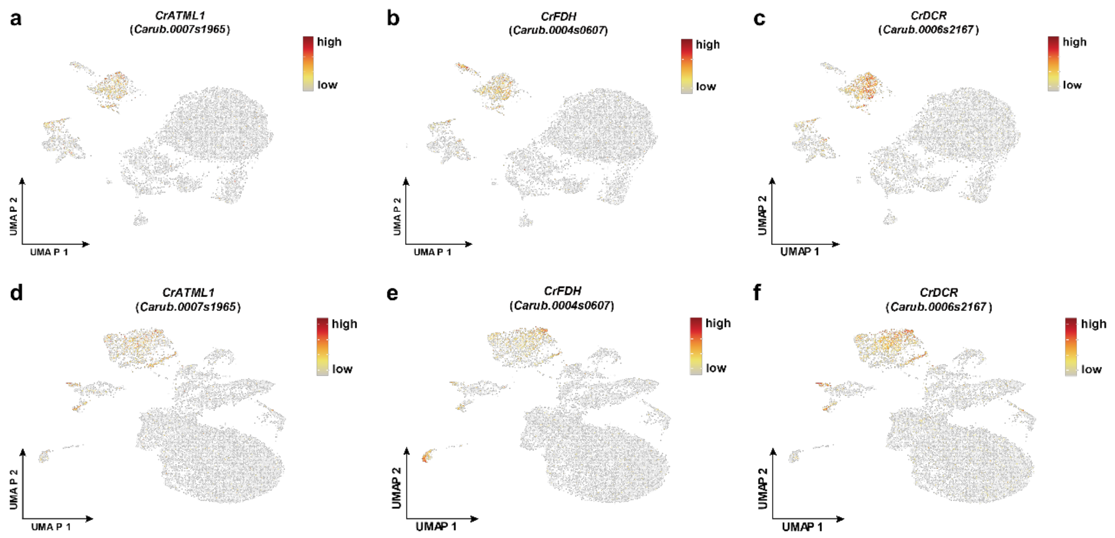

Supplementary Fig. 11 Expression of epidermal cell marker genes

a-**c**, UMAP plot showing the selected top markers genes expressed in the epidermal cells from stage 13 valve tips. **d**-**f**, UMAP plot showing the selected top markers genes expressed in the epidermal cells from stage 14 valve tips. **a** and **d**, *CrATML1*; **b** and **e**, *CrFDH*; **c** and **f**, *CrDCR*. The full name and referenced expression pattern of the selected genes are given in Supplementary Sheet 2.

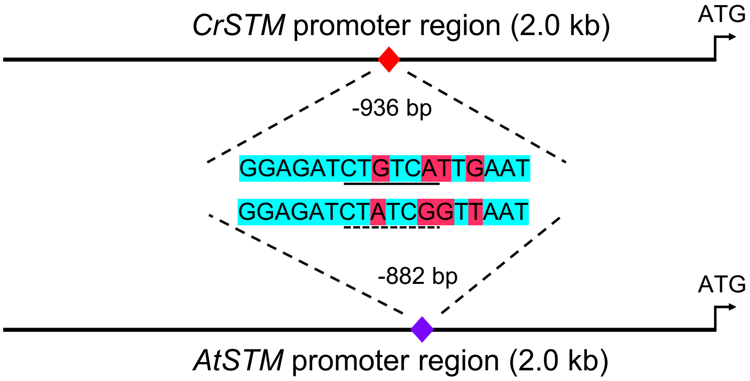

Supplementary Fig. 12 Sequence comparison of the *STM* promoters between *Capsella* and *Arabidopsis*

2000 bp sequence upstream of the transcription start site (TSS) of the STM gene was aligned between *Capsella* and *Arabidopsis*. The STM-binding site (underlined) is located at -936 bp position in the *CrSTM* promoter, which is homologous to the -882 bp region in the *AtSTM* promoters (dash line underlined). The identical sequences were shown in green and differentiated sequences were shaded in red, respectively.

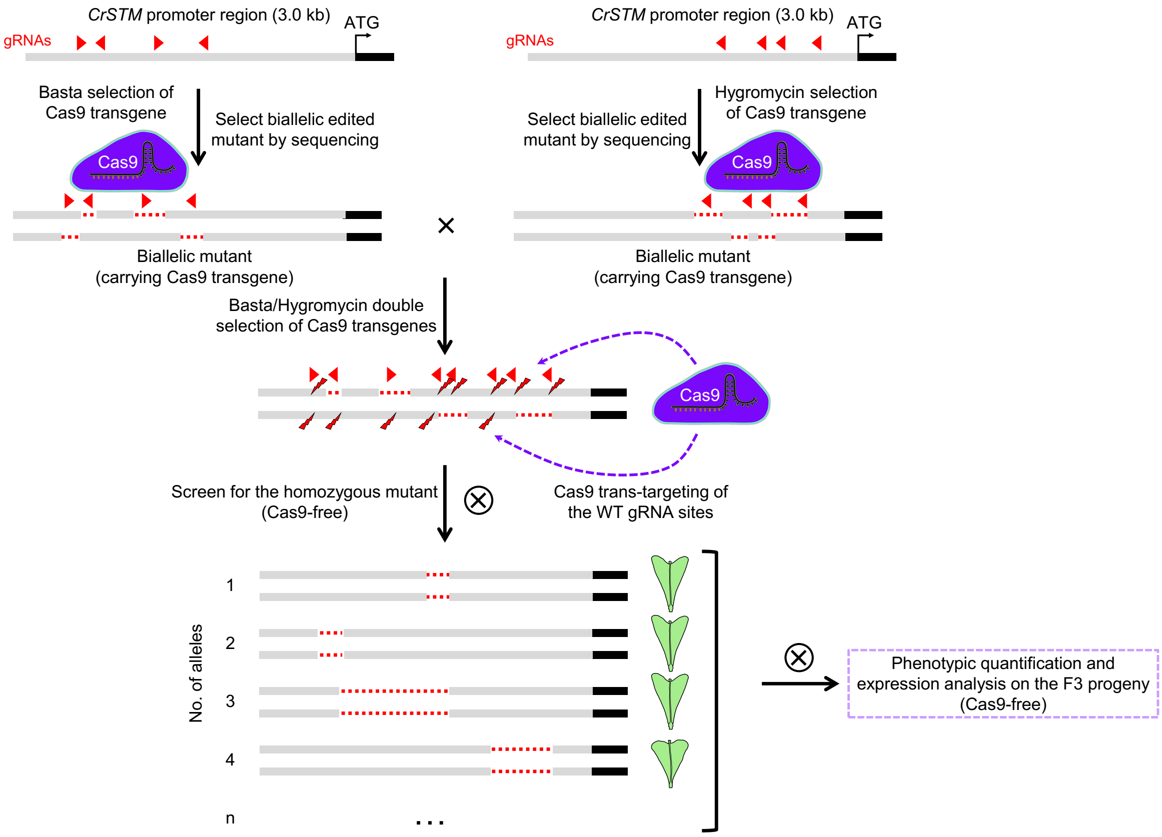

Supplementary Fig. 13 Schematic workflow generating the 22 *CrSTM* promoter mutant alleles using CRISPR/cas9.

CRISPR/Cas9 transgenic plants are generated by transforming constructs carrying two sets of 4 gRNAs targeting the CrSTM promoter. Plants carrying the Cas9 transgene are screened by Basta and hygromycin (Hyg), respectively. The positive plants are then subjected to promoter genotyping, and those biallelic for promoter mutations are then crossed. The F_1 plants inherited both Cas9 transgenes and associated 8 gRNAs (with both basta and Hyg selection marker) are screened and genotyped for biallelic mutations, confirming the effectiveness of Cas9 and gRNAs in gene editing. The F_1 plants are selfed to generate a large segregation F_2 population. The F_2 plants are then screened simultaneously against Basta and Hyg (without Cas9 transgenes) on a plate and genotyped by PCR-sequencing for homozygosity for mutant alleles. The homozygous F_2 plants of each genotype are selfed to the F_3 generation for phenotypic and expression analysis. The promoter alleles in different F_3 families are validated by PCR followed by sequencing. ATG indicates the start codon.

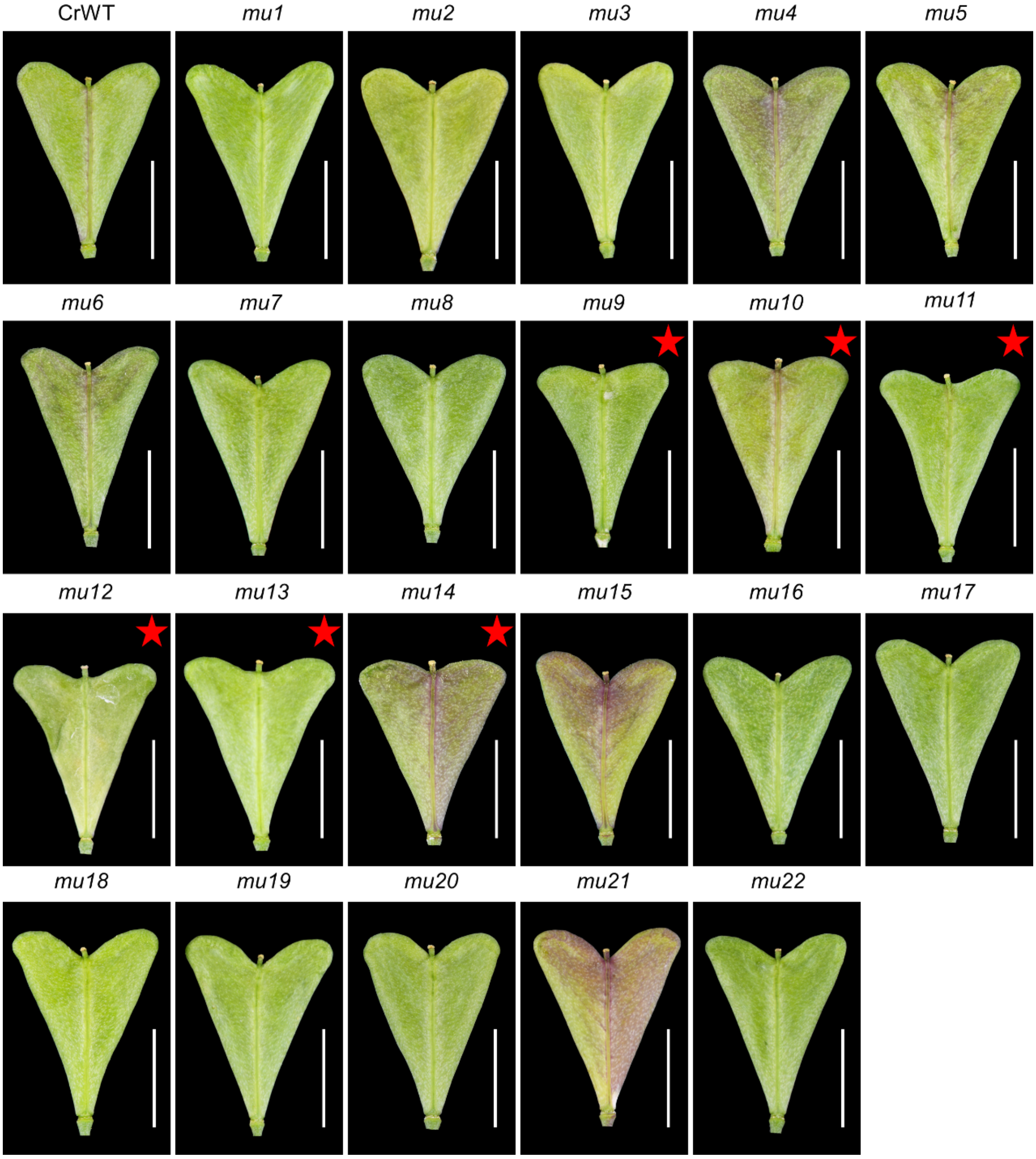

Supplementary Fig. 14 Fruit morphology of *CrSTM* promoter-edited alleles

Fruit morphology of the respective homozygous lines at stage 17. Each genotype corresponds to the details shown in Fig. 3**k**. Red stars indicate the mutant line with compromised out-growth of the fruit valve tips. Scale bars, 5 mm.

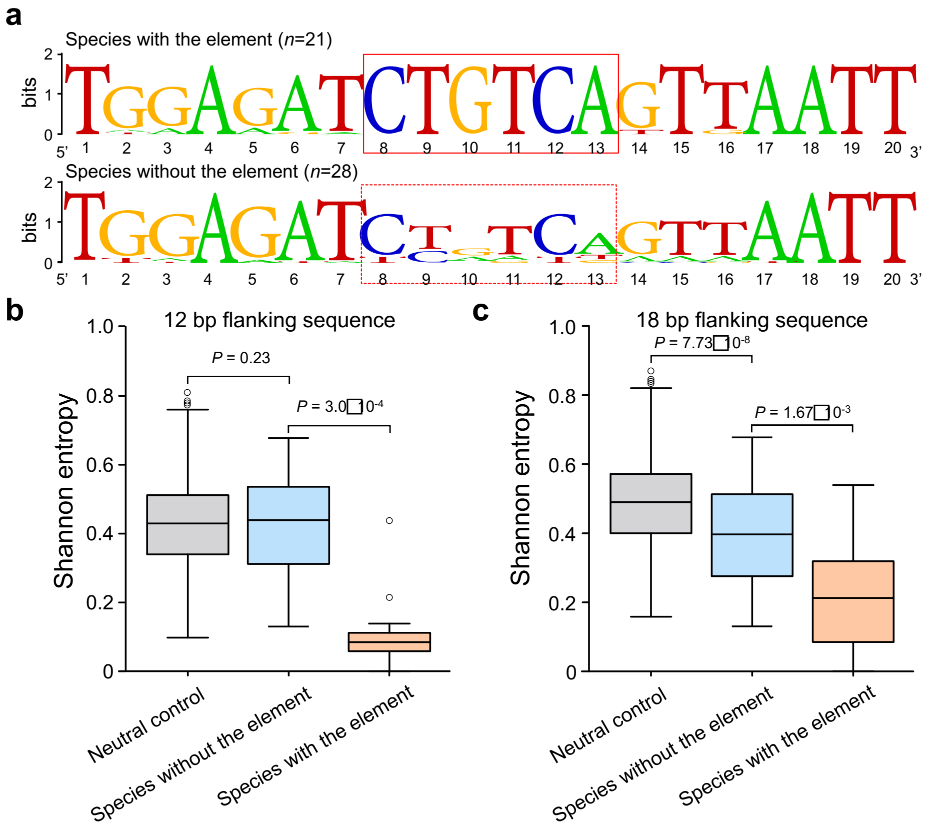

Supplementary Fig. 15 Detection of selective constraint on the STM-binding site in Brassicaceae

a, Comparison between the sequence logos of the STM-binding site from species evolved with this element (upper panel, n=21) and without this element (lower panel, n=28). Please note that the STM-binding site is 100% identical among the species that evolved with this element, while it is less conserved in the counterpart region among species without this element. The plot is generated using WebLogo online tool (https://weblogo.berkeley.edu/). **b** and **c**, Comparison of conservation at the 5' and 3' sequences flanking the STM binding site with 12-bp (**b**) and 18-bp (**c**) between groups with or without the element. Neutral control are four-fold degenerate sites (4-fold sites) from STM-orthologous genes. We generated the control group by randomly sampling six sites from all the 4-fold sites each time with 1000 repeats. In box plots of **b** and **c**, the central line indicates the median, the edges of the box define the interquartile range, and the whiskers extend to the most extreme values, excluding outliers that are depicted as open circles. *p*-values are from the two-sided Mann-Whitney U test.
