# Supplementary material for: Evolution of a SHOOTMERISTEMLESS transcription factor binding site promotes fruit shape determination
Source: Nat Plants. 2024 Dec 12;11(1):23–35. doi: 10.1038/s41477-024-01854-1 (PMC11757149; doi:10.1038/s41477-024-01854-1)
Supplement: Supplementary file 2 — Reporting Summary [file 41477_2024_1854_MOESM2_ESM.pdf]

nature portfolio

Corresponding author(s): Lars Østergaard and Yang Dong

Last updated by author(s): Jul 19, 2024

Reporting Summary

Nature Portfolio wishes to improve the reproducibility of the work that we publish. This form provides structure for consistency and transparency in reporting. For further information on Nature Portfolio policies, see our Editorial Policies and the Editorial Policy Checklist.

Statistics

For all statistical analyses, confirm that the following items are present in the figure legend, table legend, main text, or Methods section.

n/a	Confirmed	
☐	☒	The exact sample size (*n*) for each experimental group/condition, given as a discrete number and unit of measurement
☐	☒	A statement on whether measurements were taken from distinct samples or whether the same sample was measured repeatedly
☐	☒	The statistical test(s) used AND whether they are one- or two-sided<br>*Only common tests should be described solely by name; describe more complex techniques in the Methods section.*
☒	☐	A description of all covariates tested
☒	☐	A description of any assumptions or corrections, such as tests of normality and adjustment for multiple comparisons
☐	☒	A full description of the statistical parameters including central tendency (e.g. means) or other basic estimates (e.g. regression coefficient) AND variation (e.g. standard deviation) or associated estimates of uncertainty (e.g. confidence intervals)
☐	☒	For null hypothesis testing, the test statistic (e.g. *F*, *t*, *r*) with confidence intervals, effect sizes, degrees of freedom and *P* value noted<br>*Give P values as exact values whenever suitable.*
☒	☐	For Bayesian analysis, information on the choice of priors and Markov chain Monte Carlo settings
☒	☐	For hierarchical and complex designs, identification of the appropriate level for tests and full reporting of outcomes
☒	☐	Estimates of effect sizes (e.g. Cohen's *d*, Pearson's *r*), indicating how they were calculated

Our web collection on statistics for biologists contains articles on many of the points above.

Software and code

Policy information about availability of computer code

Data collection	For live imaging data collection, we used the a Zeiss inverted laser confocal microscope (Zeiss LSM 980) with ZEN Microscopy Software. The single cell RNA library was sequenced by Illumina sequencer NovaSeq 6000 plateform.
Data analysis	Data integration, clustering and annotation<br>For aligning reads and generating gene-cell matrices, the raw dataset was processed with Cell Ranger 6.0.2 (10x Genomics) software with default parameters. The nuclear genome (version v1.1), the mitochondria genome and associated GTF annotation files of Capsella rubella were downloaded from Phytozome (https://phytozome-next.jgi.doe.gov/) and NCBI (https://www.ncbi.nlm.nih.gov/), respectively. These files were subsequently combined by the "cellranger mkref" function to build the reference. Then, the gene-cell matrix was generated by the "cellranger count" function. More than 90% reads in all the samples were mapped to the reference by executing the "cellranger count" function. To test the repeatability between samples, the "cellranger aggr" function was used to merge matrices of two replicates. All the detailed information of Cell Ranger is summarized in (Extended Data Table 3). One of two replicates (Mean Reads per Cell > 40000) from the replicates was used for further analysis.<br>The resultant gene-cell matrix was then processed into Seurat (v.4.3.0) package for in-depth data analysis including quality control, normalization, dimension reduction, clustering and annotation. For quality control, we applied the following criterions: (1) cells with unique molecular identifiers (UMIs) number 2500 ~ 40000 were selected for analysis; (2) the percentage of mitochondrial UMIs was less than 5%; (3) cells containing expressed genes less than 200 were filtered out; (4) genes that were expressed in fewer than 3 cells were removed; (5) the genes with significant differential expression induced by the protoplasting process were excluded. To correct the variation caused by library preparation efficiency or sequencing depth, we used the "NormalizeData" to normalize the matrices. Top 2000 Highly Variable Genes (HVGs) were selected with the "FindVariableGenes" function by the vst method. For dimension reduction, the "RunPCA" function calculated 50 Principal Components (PCs) of HVGs and the top 20 PCs representing more than 85% accumulating contribution rate were selected for the downstream analysis. We next used the "FindNeighbors" function on the top 20 PCs to compute the nearest neighbor networks, then used

the "FindClusters" function to cluster cells with "resolution = 0.7" argument based on Louvain method. The Uniform Manifold Approximation and Projection (UMAP) method was used to visualize cell clusters. The cluster specific or preferential expressed genes were identified by the "FindAllMarkers" function with "min.pct = 0.05, logfc.threshold = 0.5". The "DotPlot" function and the "VlnPlot" function were used to define the enrichments of marker genes in the corresponding cell clusters. In the end, an additional R package "DoubletFinder" (v.2.0.2) was used to identify and remove the predicted doublet droplet from the clusters. The "plotly" (v.4.10.1) package was used to generate a 3D UMAP scatter graph.

Differentiation trajectories analysis
For reconstructing the developmental trajectory of the epidermal cells, epidermal cells (Stage-13, cluster 4, 11, 12; Stage-14, cluster 3, 12, 14) were collected. To minimize the influence of stomata lineage on trajectory reconstruction, the cells expressing FAMA were removed. In order to normalize mRNA difference between cells, the "estimateSizeFactors" function and the "estimateDispersions" function were applied. Subsequently, monocle (v.2.18.0) package was used to infer the differentiation trajectory. A semi-supervised method was used to infer the genes involved in the corresponding biology process. The core developmental steps were calculated by the differential expression genes from the initial HVGs with "differentialGeneTest" function (screening criteria, qval < 0.01). We used the DDRTree algorithm with "reduceDimension" function to reduce the dimensions. To align cells in the trajectory, the "orderCells" function was performed, the root was established by the maximum expression of ATML1, FDH, DCR in trajectory. To visualize genes involved in cell cycle and cytokinesis (Extended Data Table S4), we used the "plot genes in pseudotime" function. The number of cells expressing these genes and the expression value of corresponding genes was calculated from counts and visualized using ggplot2 (v.3.4.2) package.
For live imaging and cell behaviour analysis, MorphoGraphX was used (reference 7 of the manuscript)

For manuscripts utilizing custom algorithms or software that are central to the research but not yet described in published literature, software must be made available to editors and reviewers. We strongly encourage code deposition in a community repository (e.g. GitHub). See the Nature Portfolio guidelines for submitting code & software for further information.

Data

Policy information about availability of data

All manuscripts must include a data availability statement. This statement should provide the following information, where applicable:

- Accession codes, unique identifiers, or web links for publicly available datasets
- A description of any restrictions on data availability
- For clinical datasets or third party data, please ensure that the statement adheres to our policy

All data supporting the findings of this study are available within the paper and its Supplementary Information. The single-cell sequencing data are available in NCBI public repository under the code: PRJNA1067523

Research involving human participants, their data, or biological material

Policy information about studies with human participants or human data. See also policy information about sex, gender (identity/presentation), and sexual orientation and race, ethnicity and racism.

Reporting on sex and gender	N/A
Reporting on race, ethnicity, or other socially relevant groupings	N/A
Population characteristics	N/A
Recruitment	N/A
Ethics oversight	N/A

Note that full information on the approval of the study protocol must also be provided in the manuscript.

Field-specific reporting

Please select the one below that is the best fit for your research. If you are not sure, read the appropriate sections before making your selection.

☒ Life sciences ☐ Behavioural & social sciences ☐ Ecological, evolutionary & environmental sciences

For a reference copy of the document with all sections, see nature.com/documents/nr-reporting-summary-flat.pdf

Life sciences study design

All studies must disclose on these points even when the disclosure is negative.

Sample size	Sample-size calculation was not performed. Sample size was considered sufficient according to statistical significance of the test obtained. Typically three biological repeats were used for quantifying expression level and DNA binding, whereas 10 or more transgenic lines were analysed in phenotypic assessment.

Data exclusions	No data were excluded from the analyses.
Replication	A number between 2 and 4 biological replicates were performed and all showed reproducible results.
Randomization	The plants used in this study were grown in controlled environment rooms in random positions. Analysed samples for e.g. ChIP-PCR or qPCR were made of of tissue from different plants grown in random position.
Blinding	Blinding was not considered useful for the nature of experiments done here and logistically impossible due to geographical distance between participants. Instead we carried out biological repeats in both labs.

Reporting for specific materials, systems and methods

We require information from authors about some types of materials, experimental systems and methods used in many studies. Here, indicate whether each material, system or method listed is relevant to your study. If you are not sure if a list item applies to your research, read the appropriate section before selecting a response.

Materials & experimental systems

n/a	Involved in the study	
☐	☒	Antibodies
☒	☐	Eukaryotic cell lines
☒	☐	Palaeontology and archaeology
☒	☐	Animals and other organisms
☒	☐	Clinical data
☒	☐	Dual use research of concern
☐	☒	Plants

Methods

n/a	Involved in the study	
☒	☐	ChIP-seq
☒	☐	Flow cytometry
☒	☐	MRI-based neuroimaging

Antibodies

Antibodies used	anti-GFP antibody (Roche, no. 11814460001), concentration: 0.4 µg/µl.
Validation	Commercially available. Hela cells WCL has been loaded on a 4-12% SDS-precast gel (1:30h; 180 V) then proteins have been transferred on nitrocellulose membrane using the semi-dry blotting (1h 12V). Blocking with milk 5% in PBS 0,05% tween (45 minutes) was followed by incubation overnight at room temperature with anti- GFP antibody (1:2000 in milk 5% in PBS 0,05% tween). Then the membrane was washed with 3x 5 minutes with PBS 0,05% tween, followed by a 45 minutes incubation with Mouse secondary antibody 1:3000 in milk 5% in PBS 0,05% tween. The secondary antibody was washed out with a second wash with PBS 0,05% tween (3x 5 minutes). ECL substrate has been used to detect the signal.

Dual use research of concern

Policy information about dual use research of concern

Hazards

Could the accidental, deliberate or reckless misuse of agents or technologies generated in the work, or the application of information presented in the manuscript, pose a threat to:

No	Yes	
☒	☐	Public health
☒	☐	National security
☒	☐	Crops and/or livestock
☒	☐	Ecosystems
☒	☐	Any other significant area

Experiments of concern

Does the work involve any of these experiments of concern:

No	Yes	
☒	☐	Demonstrate how to render a vaccine ineffective
☒	☐	Confer resistance to therapeutically useful antibiotics or antiviral agents
☒	☐	Enhance the virulence of a pathogen or render a nonpathogen virulent
☒	☐	Increase transmissibility of a pathogen
☒	☐	Alter the host range of a pathogen
☒	☐	Enable evasion of diagnostic/detection modalities
☒	☐	Enable the weaponization of a biological agent or toxin
☒	☐	Any other potentially harmful combination of experiments and agents
