# Supplementary material for: Evolution of a SHOOTMERISTEMLESS transcription factor binding site promotes fruit shape determination
Source: Nat Plants. 2024 Dec 12;11(1):23–35. doi: 10.1038/s41477-024-01854-1 (PMC11757149; doi:10.1038/s41477-024-01854-1)
Supplement: Supplementary file 3 — List of the Brassicaceae species used in this study. [file 41477_2024_1854_MOESM3_ESM.pdf]

Extended Data Table 1. List of Brassicaceae species used in this study

Taxon	Sample accessions	Experiment	Collection location	Sequence acquisition
Arabidopsis thaliana	Bra-82	a, b, c	N/A	PCR-Sequencing
Arabidopsis halleri	Bra-143	a, b	N/A	Phytozome* (Araha1654s0002)
Meniocus linifolius	Bra-68	a	Changji, Xinjiang	TAIL-PCR-Sequencing
Barbarea orthoceras	Bra-3	a, b	Baishan, Jilin	TAIL-PCR-Sequencing
Berteroa incana	Bra-4	a, b	N/A	TAIL-PCR-Sequencing
Brassica rapa	Bra-78	a	N/A	Phytozome* (BraraH01745)
Bunias orientalis	Bra-139	a, c	Beijing, Beijing	TAIL-PCR-Sequencing
Camelina sativa	Bra-5	a, b	Altay, Xinjiang	TAIL-PCR-Sequencing
Capsella grandiflora	Bra-144	a, b	N/A	Phytozome* (Cagra5414s0060)
Capsella rubella	Bra-145	a, b, c	N/A	PCR-Sequencing
Cardamine circaeoides	Bra-59	a, b	N/A	TAIL-PCR-Sequencing
Cardamine hirsuta	Bra-58	a, b	Beijing, Beijing	TAIL-PCR-Sequencing
Chorispora tenella	Bra-7	a	Harbin, Heilongjiang	TAIL-PCR-Sequencing
Christolea crassifolia	Bra-8	a, b	N/A	TAIL-PCR-Sequencing
Crambe hispanica	Bra-70	a, b	N/A	TAIL-PCR-Sequencing
Dilophia salsa	Bra-15	a	Kashi, Xinjiang	TAIL-PCR-Sequencing
Diplotaxis muralis	Bra-16	a, b	Jilin, Jilin	TAIL-PCR-Sequencing
Draba draboides	Bra-11	a, b	Haibei, Qinghai	TAIL-PCR-Sequencing
Draba lanceolata	Bra-19	a, b	Ili, Xinjiang	TAIL-PCR-Sequencing
Eruca vesicaria	Bra-20	a, c	Aba, Sichuan	TAIL-PCR-Sequencing
Euclidium syriacum	Bra-22	a, b	Urumqi, Xinjiang	Phytozome* (Eusyr0133s0289)
Eutrema altaicum	Bra-47	a, b, c	Ili, Xinjiang	TAIL-PCR-Sequencing

Extended Data Table 1. List of Brassicaceae species used in this study (continued)

Taxon	Sample accessions	Experiment	Collection location	Sequence acquisition
Eutrema salsugineum	Bra-50	a, b	Jinan, Shandong	Phytozome* (Thhalv10024934m)
Goldbachia pendula	Bra-24	a, c	Tacheng, Xinjiang	TAIL-PCR-Sequencing
Hesperis sibirica	Bra-26	a, b	Chengde, Hebei	TAIL-PCR-Sequencing
Iberis amara	Bra-53	a, b	N/A	Phytozome* (Ibeam2899s0006)
Isatis multicaulis	Bra-36	a	Changji, Xinjiang	TAIL-PCR-Sequencing
Isatis violascens	Bra-27	a, b	Changji, Xinjiang	TAIL-PCR-Sequencing
Lepidium apetalum	Bra-64	a, b	N/A	TAIL-PCR-Sequencing
Lepidium didymum	Bra-60	a, b, c	N/A	TAIL-PCR-Sequencing
Lepidium draba	Bra-88	a, c	Tacheng, Xinjiang	TAIL-PCR-Sequencing
Leptaleum filifolium	Bra-29	a, b	Karamay, Xinjiang	TAIL-PCR-Sequencing
Malcolmia scorpioides	Bra-30	a, b	Changji, Xinjiang	TAIL-PCR-Sequencing
Nasturtium officinale	Bra-65	a, b	N/A	TAIL-PCR-Sequencing
Neslia paniculata	Bra-33	a, b	Ili, Xinjiang	TAIL-PCR-Sequencing
Parrya nudicaulis	Bra-37	a	Ili, Xinjiang	TAIL-PCR-Sequencing
Rorippa palustris	Bra-125	a, b, c	Ili, Xinjiang	TAIL-PCR-Sequencing
Rudolf-kamelinia korolkowii	Bra-32	a, b, c	Bortala, Xinjiang	TAIL-PCR-Sequencing
Schrenkiella parvula	Bra-77	a, b	N/A	Phytozome* (Sp7g35580)
Sisymbriopsis mollipila	Bra-41	a, b	Kashi, Xinjiang	TAIL-PCR-Sequencing
Solms-laubachia eurycarpa	Bra-44	a, b	Haixi, Qinghai	TAIL-PCR-Sequencing
Thlaspi arvense	Bra-66	a, b	Shangri-La, Yunnan	Phytozome* (Thlar0005s0217)
Yinshania henryi	Bra-57	a, b	Duyun, Guizhou	TAIL-PCR-Sequencing
Cleome violacea	N/A	a	N/A	Phytozome* (Clevi0022s0646)

Extended Data Table 1. List of Brassicaceae species used in this study (continued)

Taxon	Sample accessions	Experiment	Collection location	Sequence acquisition
Crucihimalaya himalaica	Bra-14	b	N/A	TAIL-PCR-Sequencing
Diptychocarpus strictus	Bra-17	b	Urumqi, Xinjiang	TAIL-PCR-Sequencing
Draba yunnanensis	Bra-102	b	N/A	TAIL-PCR-Sequencing
Erysimum canescens	Bra-104	b	Changji, Xinjiang	TAIL-PCR-Sequencing
Erysimum cheiranthoides	Bra-105	b	Shijiazhuang, Hebei	TAIL-PCR-Sequencing
Erysimum siliculosum	Bra-21	b	Changji, Xinjiang	TAIL-PCR-Sequencing
Erysimum wardii	Bra-107	b	N/A	TAIL-PCR-Sequencing
Smelowskia tibetica	Bra-25	b	Haibei, Qinghai	TAIL-PCR-Sequencing
Isatis costata	Bra-109	b	Altay, Xinjiang	TAIL-PCR-Sequencing
Lunaria annua	Bra-74	b	N/A	Phytozome* (Luann0043s0143)
Noccaea thlaspidioides	Bra-135	b	Altay, Xinjiang	TAIL-PCR-Sequencing
Solms-laubachia parryoides	Bra-38	b	Gannan, Gansu	TAIL-PCR-Sequencing
Lepidium chalepense	Bra-6	b	Karamay, Xinjiang	TAIL-PCR-Sequencing
Rorippa cantoniensis	Bra-55	b	N/A	TAIL-PCR-Sequencing
Rorippa elata	Bra-123	b	Aba, Sichuan	TAIL-PCR-Sequencing
Smelowskia sisymbrioides	Bra-45	b	Ili, Xinjiang	TAIL-PCR-Sequencing

Experiment details, a, Phylogeny and STM-binding site origin; b, Molecular evolution analysis of STM-binding site; c, SEM analysis of gynoecium development.
*These sequences were downloaded from Phytozome (https://phytozome-next.jgi.doe.gov/) with a unique gene code in the parentheses.
