# Supplementary material for: Evolution of a SHOOTMERISTEMLESS transcription factor binding site promotes fruit shape determination
Source: Nat Plants. 2024 Dec 12;11(1):23–35. doi: 10.1038/s41477-024-01854-1 (PMC11757149; doi:10.1038/s41477-024-01854-1)
Supplement: Supplementary file 4 — List of the oligonucleotides used in this study. [file 41477_2024_1854_MOESM4_ESM.pdf]

Extended Data Table 2. List of oligonucleotides used in this study

Primer	Sequence (5' to 3')	Used in experiment
pCrSTM (2.0 kb)-F	*CCTCTAGAGTCGACCTGCAGGAGAGTTTTATTTTCTTCTTTC	GUS reporter construction
pCrSTM (1.5 kb)-F	*CCTCTAGAGTCGACCTGCAGAAAACAGGTGTCCTATATTC	GUS reporter construction
pCrSTM (1.0 kb)-F	*CCTCTAGAGTCGACCTGCAGTTTCCTATTTGACAAAAGAG	GUS reporter construction
pCrSTM (0.9kb)-F	*CCTCTAGAGTCGACCTGCAGAAGTTTTTTGGGTTTTTTTGTA	GUS reporter construction
pCrSTM (0.8kb)-F	*CCTCTAGAGTCGACCTGCAGTTTTTAACCAACATATACGTAT	GUS reporter construction
pCrSTM (0.7kb)-F	*CCTCTAGAGTCGACCTGCAGCAAAAGAAAGACCAACAAATTA	GUS reporter construction
pCrSTM (0.6kb)-F	*CCTCTAGAGTCGACCTGCAGTCAATATAAACTGAGATCCGGT	GUS reporter construction
pCrSTM (0.5kb)-F	*CCTCTAGAGTCGACCTGCAGCACAAAAGAGTTAAGAACCC	GUS reporter construction
pCrSTM -R	*CTCAGATCTACCATGGCTTCTCTTTCTCTCACTAGTATTATTATTC	GUS reporter construction
pCrWUS-F	*CCTCTAGAGTCGACCTGCAGAGAACAAATGCCAAATTAAAAG	GUS reporter construction
pCrWUS-R	*CTCAGATCTACCATGGGTGTGTTTGCTTAGAGAAAAAT	GUS reporter construction
pCrKNAT2-F	*CCTCTAGAGTCGACCTGCAGATCACTTTTGTTGTTGAACAGA	GUS reporter construction
pCrKNAT2-R	*CTCAGATCTACCATGGAAGTATGAATATGATACGTCTC	GUS reporter construction
pCrKNAT6-F	*CCTCTAGAGTCGACCTGCAGGTGAGTAAGTCAGCTGATACTT	GUS reporter construction
pCrKNAT6-R	*CTCAGATCTACCATGGAAGTATGAATATGATACGTCTC	GUS reporter construction
pCrCYCB1;1-F	*CCTCTAGAGTCGACCTGCAGCAAAATCCAACATGGCAGTA	GUS reporter construction
pCrCYCB1;1-R	*CTCAGATCTACCATGGCTATGCCTTCGCTATCTCTC	GUS reporter construction
pCrCYCB1;2-F	*CCTCTAGAGTCGACCTGCAGAATGGTTTTGATGGACAATG	GUS reporter construction
pCrCYCB1;2-R	*CTCAGATCTACCATGGGATCGCTCTCCTACTGAATC	GUS reporter construction
pCrSTM:gCrSTM-F	*ATCCTCTAGAGTCGACTGAAGGCAGTGGCTGAGGAGAG	GFP-tagged reporter construction
pCrSTM:gCrSTM-R	*GTCAGATCTACCATGGAAAGCATGGTGGATGAGATG	GFP-tagged reporter construction

Extended Data Table 2. List of oligonucleotides used in this study (continued)

**Primer**	**Sequence (5' to 3')**	**Used in experiment**
pCrARF6:gCrARF6-F	*ATCCTCTAGAGTCGACACGGATTCTTGAATGATGAAAG	GFP-tagged reporter construction
pCrARF6:gCrARF6-R	*GTCAGATCTACCATGGAGTAGTTGAATGTACCCCCTACTG	GFP-tagged reporter construction
pCrARF8:gCrARF8-F	*ATCCTCTAGAGTCGACTATAGGGAGTATGTTAGTTAGG	GFP-tagged reporter construction
pCrARF8:gCrARF8-R	*GTCAGATCTACCATGGAGAGATGGGTTGGGTTTTGCGG	GFP-tagged reporter construction
pAtSTM:gAtSTM-F	*ATCCTCTAGAGTCGACCTTCGTACAATCCTCCAAATTGAC	GFP-tagged reporter construction
pAtSTM:gAtSTM-R	*GTCAGATCTACCATGGGCATGGTGGAGGAGATGTGA	GFP-tagged reporter construction
35S:CrSTM:GR-F	*GCCCAAGCTACGCGTCATGGAGAGTGGTTCCAACAG	DEX-inducible line construction
35S:CrSTM:GR-R	*ATCAGCTCCGGCGCCGCAAAGCATGGTGGATGAGA	DEX-inducible line construction
pLhGR>> CrSTM-F	¶T**GAAGAC**TTAATGGAGAGTGGTTCCAACAGC	DEX-inducible line construction
pLhGR>> CrSTM-R	¶T**GAAGAC**TTAAGCAAGCATGGTGGATGAGATGTG	DEX-inducible line construction
miR319-Backbone-F	ACAAACACACGCTCGGACG	microRNA319 isolation
miR319-Backbone-R	CATGGCGATGCCTTAAATAAAG	microRNA319 isolation
pLhGR>>amiR-CrSTM-F	¶T**GAAGAC**TTAATGACAAACACACGCTCGGACG	DEX-inducible line construction
pLhGR>>amiR-CrSTM-R	¶T**GAAGAC**TTAAGCCATGGCGATGCCTTAAATAAAG	DEX-inducible line construction
CrSTM-Exon1-gRNA1	TGATGTGATGTCATGATGGG	Gene editing
CrARF6-Exon2-gRNA1	CTTGTCTCGTTACCTCCTGT	Gene editing
CrARF8-Exon1-gRNA1	ACATCTGGATTGGGTCAACA	Gene editing
CrSTM-promoter-gRNA1	CTACGTTCACTTCAGTCAAG	Gene editing
CrSTM-promoter-gRNA2	CAAATACTCTGAAAGCAGTA	Gene editing
CrSTM-promoter-gRNA3	AGAAATGGCAGTGAAGGCAG	Gene editing
CrSTM-promoter-gRNA4	TTAACAAACAAAAATAGCAT	Gene editing
CrSTM-promoter-gRNA5	CATTTTAGAGTTAATTTACG	Gene editing

Extended Data Table 2. List of oligonucleotides used in this study (continued)

**Primer**	**Sequence (5' to 3')**	**Used in experiment**
CrSTM-promoter-gRNA6	ACATCTATATAGGTTTTTGA	Gene editing
CrSTM-promoter-gRNA7	GATGGGATATACGTATATGT	Gene editing
CrSTM-promoter-gRNA8	CTAAATAGTGATAGTTATGG	Gene editing
CrSTM-Exon1-Geno-F	GGAGAGTGGTTCCAACAGCAC	Gene CRISPR genotyping
CrSTM-Exon1-Geno-R	CAATTGACATAAGCGGCCAAG	Gene CRISPR genotyping
CrARF6-Exon2-Geno-F	TTTGTTTGGATTCAAAGTTAC	Gene CRISPR genotyping
CrARF6-Exon2-Geno-R	CATCAGCCTGAAAATGTAAAG	Gene CRISPR genotyping
CrARF8-Exon2-Geno-F	GGTTTTCGAAAAGTTTGTGAC	Gene CRISPR genotyping
CrARF8-Exon2-Geno-R	TATTGTGCACACCAGTGGAGG	Gene CRISPR genotyping
pCrSTM-gRNA1/2-Geno-F	GCTTAAAACCCTAAATAATG	Promoter CRISPR genotyping
pCrSTM-gRNA1/2-Geno-R	TTACCATCCAAATTTTAACT	Promoter CRISPR genotyping
pCrSTM-gRNA3/4-Geno-F	AAATCGAGTGTTCGGAAAAT	Promoter CRISPR genotyping
pCrSTM-gRNA3/4-Geno-R	CTATTAGCAATTGTTAGCAC	Promoter CRISPR genotyping
pCrSTM-gRNA5-Geno-F	CCTTCTTCAAATCCACAATGAC	Promoter CRISPR genotyping
pCrSTM-gRNA5-Geno-R	ACGGCTTGTTAATTTAGACACC	Promoter CRISPR genotyping
pCrSTM-gRNA6-Geno-F	CGTTTCTAGAGGTTGAACTA	Promoter CRISPR genotyping
pCrSTM-gRNA6-Geno-R	TGGACAGAGATTGTGTGATC	Promoter CRISPR genotyping
pCrSTM-gRNA7/8-Geno-F	CTGTCATTGAATTATTGAGAATC	Promoter CRISPR genotyping
pCrSTM-gRNA7/8-Geno-R	TGAAAATGCAAAAGTGGTCTCC	Promoter CRISPR genotyping
CrSTM-3'UTR-qPCR-F	CTTTCCCCATGGATCACATCTC	Gene expression analysis
CrSTM-3'UTR-qPCR-R	TAAACGCTTGTTCATCAATCGG	Gene expression analysis
amiR-CrSTM-qPCR-F	CAAGTCTTGCGCATGTACAGT	Gene expression analysis
amiR-CrSTM-qPCR-R	CAGGTCTTGCGCAAGTACAGA	Gene expression analysis

Extended Data Table 2. List of oligonucleotides used in this study (continued)

**Primer**	**Sequence (5' to 3')**	**Used in experiment**
CrCDKB1;1-qPCR-F	GTGTTTCCACTCTCCGTGACTG	Gene expression analysis
CrCDKB1;1-qPCR-R	TTCGGCTGGATTGTACTTGAGC	Gene expression analysis
CrCYCD3;2-qPCR-F	GTGTGAGCGTCTTCTGATCTCC	Gene expression analysis
CrCYCD3;2-qPCR-R	TGCTCCAACAACAGTTCGTAGC	Gene expression analysis
CrCYCA1;1-qPCR-F	CAAGTCTGCGAAATTGAAGTTG	Gene expression analysis
CrCYCA1;1-qPCR-R	CCATCATCAGATTTACTAGGAG	Gene expression analysis
CrCYCB2;1-qPCR-F	GAGAGCTTTAGCTGTGTTCCAG	Gene expression analysis
CrCYCB2;1-qPCR-R	CACTGTGAGAAACAGTGTCTCG	Gene expression analysis
CrCYCB1;1-qPCR-F	CCACATACACATCTGTTCTTAC	Gene expression analysis
CrCYCB1;1-qPCR-R	TGCATGTAATCGCTTGGTCTCC	Gene expression analysis
CrCYCB1;2-qPCR-F	GTGATCGAGACAAAGAAGGAGG	Gene expression analysis
CrCYCB1;2-qPCR-R	TATGCCACACGCAGCTTTGCTC	Gene expression analysis
CrSTM:GFP-ChIP-A-F	CAGATTATATGCTCTGACTTG	ChIP-qPCR
CrSTM:GFP-ChIP-A-R	TAGGATTTAAGAGAATCTATCG	ChIP-qPCR
CrSTM:GFP-ChIP-B-F	ATTTGTAAATTTTATCTTGCCTG	ChIP-qPCR
CrSTM:GFP-ChIP-B-R	ACACTCGATTTTTTTTACTAGCTTC	ChIP-qPCR
CrSTM:GFP-ChIP-C-F	CTAAAGAGTCTTATCCTTTCCTATT	ChIP-qPCR
CrSTM:GFP-ChIP-C-R	ACAAAAAAACCCAAAAAACTTTC	ChIP-qPCR
CrSTM:GFP-ChIP-D-F	GACCACTTTTGCATTTTCATAT	ChIP-qPCR
CrSTM:GFP-ChIP-D-R	ACTACAAGTGTATTGGGAAACC	ChIP-qPCR
CrARF6/8:GFP-ChIP-A-F	CTTACAGAGCATTTACTTGACT	ChIP-qPCR
CrARF6/8:GFP-ChIP-A-R	CAATCATATGTTGATATATGTC	ChIP-qPCR

Extended Data Table 2. List of oligonucleotides used in this study (continued)

Primer	Sequence (5' to 3')	Used in experiment
CrARF6/8:GFP-ChIP-B-F	CAAGTGGAGACTACAAATTGAG	ChIP-qPCR
CrARF6/8:GFP-ChIP-B-R	ATTACTAAAAAGCGATTTTGGA	ChIP-qPCR
CrARF6/8:GFP-ChIP-C-F	GAAGCTAGTAAAAAAAATCGAG	ChIP-qPCR
CrARF6/8:GFP-ChIP-C-R	GCAAAGAAAAGATAACTGAGAA	ChIP-qPCR
CrARF6/8:GFP-ChIP-D-F	TACATGAATACATACACGTACA	ChIP-qPCR
CrARF6/8:GFP-ChIP-D-R	ATTTGGTATAGTTTTTTTCATA	ChIP-qPCR
CrARF6/8:GFP-ChIP-E-F	CTCTGTCCATGTCTGCTTTTTC	ChIP-qPCR
CrARF6/8:GFP-ChIP-E-R	ATAGTTATGGGGGAGAGGGAAG	ChIP-qPCR

*The sequences shaded in yellow indicate the homologs to the plasmid and used for In-Fusion cloning.

¶The *Bpi*I restriction site for the golden-gate cloning was indicated by bold letters.
